# Characterisation of human in vitro tumour-associated macrophage models to define translational relevance

**DOI:** 10.1038/s41598-025-30224-w

**Published:** 2025-11-28

**Authors:** Arthur Dyer, Rebecca Dudley, Shreya Ahuja, Clara Alsinet, Pawan Poudel, Georgina Bowyer, Kalvin Sahota, Saly Songvilay, Des C. Jones, Matthew Glover, Sonja Hess, Elina Timosenko, Simon J. Dovedi

**Affiliations:** 1 Early Oncology, R&D, AstraZeneca, Cambridge, UK; 2 Dynamic Omics, Centre for Genomics Research, Discovery Sciences, R&D, AstraZeneca, Gaithersburg, MD USA; 3 Oncology Bioinformatics, AstraZeneca, Barcelona, Spain; 4 Present Address: Immunocore, Oxford, UK; 5 Oncology Bioinformatics, AstraZeneca, Cambridge, UK

**Keywords:** Cancer, Cancer microenvironment, Cancer models, Tumour immunology, Immunology, Immune evasion, Innate immune cells, Oncology, Cancer

## Abstract

Tumour-associated macrophages (TAMs) are key components of the tumour microenvironment with a demonstrated ability to modulate anti-tumour T-cell responses and immunotherapy outcomes. With increasing realisation that the M1/M2 paradigm does not reflect the complexity of macrophage phenotypes in cancer patients, an urgent need has arisen to develop improved, translatable in vitro models for human TAMs. To address this gap, we have screened conditioned media from a panel of tumour cell lines for their ability to induce suppressive marker upregulation on human monocyte-derived macrophages, as well as active T-cell immunosuppression. We performed secretome characterization of these tumour-conditioned media (TCM) to shed light on cancer cell-derived soluble factors that may contribute to TAM polarisation. Furthermore, we characterized the proteomic and transcriptomic signatures of macrophages exposed to either TCM or primary ascites fluid from ovarian cancer patients and performed bioinformatics analysis to determine the most translationally relevant models of TAMs. In summary, our work provides mechanistic insights on tumour-macrophage crosstalk in the context of establishing suppressive TAM phenotypes and addresses the long-standing gap of defining translationally relevant human in vitro TAM models.

**Supplementary Information:**

The online version contains supplementary material available at 10.1038/s41598-025-30224-w.

## Introduction

Macrophages are immune cells that exhibit remarkable versatility. Their ability to respond to a wide array of signals by adopting different functional phenotypes is crucial for resolving inflammation and promoting tissue repair^1^. For instance, upon encountering pathogens, macrophages assume antimicrobial functions, which are characterised by the secretion of pro-inflammatory cytokines such as IL-12 and the production of nitric oxide via nitric oxide synthase 2 (NOS2)^2^. Conversely, following tissue injury, macrophages promote repair and immunoregulation by responding to cytokines such as IL-4 and IL-13 produced primarily by T helper 2 cells, and by metabolising arginine via arginase-1 into ornithine, which inhibits T-cell proliferation^3–5^.

Inspired by the established nomenclature of the T helper (Th) 1 versus Th2 cell phenotypes, the extremes of macrophage polarisation have traditionally been categorised into "M1″ or "M2″^4^. In reality, macrophage plasticity is not a binary state but is spectrum of activation states influenced by the local microenvironment^6–8^. The traditional M1/M2 paradigm, while useful for in vitro studies, oversimplifies the complexity observed in vivo, where macrophages exhibit a continuum of states shaped by factors including cytokines, growth factors, and availability of nutrients and oxygen. Tumour-associated macrophages (TAMs) are implicated in every stage of tumour progression, from neoplastic transformation to metastasis^9^. During early tumourigenesis, the activation of oncogenes and release of danger-associated molecular patterns can activate macrophages in an M1-like fashion, triggering the production of tumour necrosis factor-alpha (TNF-α), reactive oxygen species, and nitric oxide^10^. Although these factors can be tumouricidal, they also create a mutagenic environment that may accelerate neoplastic transformation^11,12^. As tumours progress, TAMs are educated by tumour-derived signals such as IL-4, IL-10, IL-13, CSF-1, and lactic acid, towards a more M2-like phenotype^7,10,13,14^. Tumour-educated TAMs can then secrete IL-10, TGF-β, and regulatory T-cell (Treg)-attracting chemokines such as CCL22, further promoting immunosuppression in the adaptive compartment^15,16^. Malignant ascites in certain cancers further complicates this landscape, providing a unique microenvironment rich in tumour cells, non-cancerous cells, and soluble factors that promote tumour progression and chemoresistance.

Given the multifaceted role of TAMs in modulating tumour-infiltrating lymphocyte (TIL) function and the tumour microenvironment (TME), targeting TAMs has emerged as a promising therapeutic strategy. Approaches such as reprogramming TAMs towards a pro-inflammatory phenotype, depleting TAMs, or blocking their recruitment to the tumour, are being explored in pre-clinical and clinical settings. These therapeutic strategies aim to alleviate the immunosuppression and, in turn, enhance the efficacy of TILs, improving patient outcomes.

Approximately 30% of pharmaceuticals under development fail in human clinical trials due to adverse reactions, while an additional 60% fail due to lack of efficacy^17–19^. This substantial failure rate, incurring an estimated annual cost of $50–$60 billion, is partly attributed to the inadequate predictive accuracy of pre-clinical models^20^. A recent review article analysing the current clinical landscape of macrophage-reprogramming cancer immunotherapies highlighted an increase in TAM-targeted therapies entering clinical trials. Notably, 100% of IDO1 inhibitors, 61% of TLR agonists and 50% of STAT-targeted clinical trials have been discontinued despite promising in vitro results. Bridging the gap between laboratory research and clinical efficacy is therefore crucial for improving the success rate of new treatments. This involves developing translationally relevant pre-clinical models, especially for highly plastic cells like macrophages that are heavily influenced by their microenvironment. Most current research modelling TAMs in vitro predominantly relies upon either healthy donor monocytes, or the monocytic cell line THP-1 polarised, using recombinant cytokines. A significant body of work has emerged suggesting THP-1 cells do not behave similarly to primary macrophages. Consequently, translating findings from THP-1 cells or similar simplistic models to clinical applications presents challenges (reviewed in Mohd Yasin et al. 2022)^22^.

Our study attempts to mimic tumour-derived signals that drive TAM polarisation in vivo by using a panel of TCM and primary ascites fluids. Leveraging advanced proteomics technologies and bioinformatics tools, we analyse the secretome of TCM and the proteomic and transcriptomic landscape of macrophages exposed to these signals. By investigating the tumour-macrophage crosstalk at a molecular level, we seek to address the long-standing gap of defining translationally relevant, and reliably immunosuppressive human in vitro TAM models to help advance the development of more effective cancer treatments in the future. The scope of this paper encompasses the characterisation of in vitro TAM polarisation models to critically assess their translational relevance for preclinical research and does not seek to identify precise factors are responsible for TAM polarisation in vitro; such mechanistic dissection of the underlying signalling pathways is beyond the scope of this manuscript.

## Results

### Phenotypic analysis of hMDMs polarised in a panel of tumour-conditioned media

**Fig. 1:**
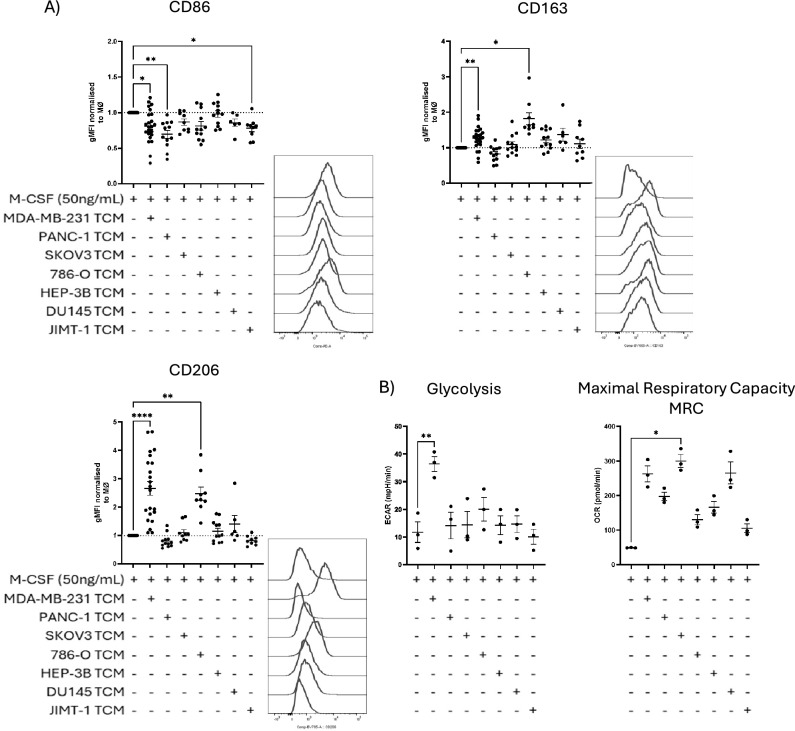
Phenotypic characterisation of hMDMs exposed to TCM. All monocytes were isolated from healthy donor PBMCs and differentiated with M-CSF for 7 days with M-CSF +/− TCM (1:1 TCM:RPMI) as indicated in the figure below before analysis. (**A**) Flow cytometry assessment of CD86, CD163 and CD206 surface marker expression demonstrating the effect of TCM exposure throughout differentiation on macrophage phenotype. Values normalised with respect to MØ (50 ng/mL M-CSF). Representative donor histograms shown. (**B**) Seahorse metabolic flux assessment on TCM-treated macrophages, Glycolysis and Maximal Respiratory Capacity shown. Each point represents one donor, values shown as Mean +/− SEM. Statistical significance calculated by Mixed effects analysis with Dunnet’s multiple comparisons test (**A**) or one-way ANOVA with Šídák's multiple comparisons test (**B**) and shown where *p-value < 0.05, **p-value < 0.005 and ***p-value < 0.0005 and ****p-value < 0.0001.

To polarise human healthy donor monocyte-derived macrophages (hMDMs) towards a TAM-like phenotype, we exposed isolated monocytes to TCM from a panel of tumour cell lines supplemented with M-CSF (Table S1). We first analysed the expression of macrophage cell surface markers using flow cytometry (Fig. 1A, S1). TCM from the breast cancer tumour cell line MDA-MB-231 led to an increase in CD163 (*p* = 0.0053) & CD206 (*p* < 0.0001). TCM from the renal cell carcinoma cell line 786-O also increased CD206 (*p* = 0.0028) & CD163 (*p* = 0.0109). Macrophages polarised with IL-4/IL-13 also showed an upregulation of CD163 (*p* = 0.0156) and albeit non-significantly, indicated a similar trend for CD206 expression (Fig. S2A). The other TCM did not significantly affect the expression of these markers. None of the conditions increased the CD86 above hMDMs polarised using M-CSF alone (M0 macrophages). hMDMs polarised with PANC-1, MDA-MB-231 and JIMT-1 TCM did lead to a significant decline in CD86 (*p* = 0.0024, 0.0102 & 0.0216, respectively).

In vitro, polarised macrophages and M0 macrophages have previously been reported to have distinct metabolic profiles^17^. We performed Seahorse metabolic flux analysis to study the extracellular acidification rate (ECAR) and oxygen consumption rate (OCR, Fig. 1B, S3). The Mito Stress Test revealed that MDA-MB-231 TCM macrophages had the highest rate of glycolysis (*p* = 0.0019). Although not as high as MDA-MB-231 TCM treatments, 786-O treated macrophages appeared to have increased glycolytic activity over other TCM. SKOV-3 (*p* = 0.0254) macrophages had the greatest maximal respiratory capacity (MRC).

These data demonstrate that culturing hMDMs with TCM, particularly from MDA-MB-231 and 786-O cells, results in upregulation of TAM-like markers and an altered metabolic profile. This suggests that specific tumour-derived factors can drive macrophage polarisation towards a suppressive TAM-like phenotype.

### hMDMs polarised with MDA-MB-231 tumour-conditioned media inhibit proliferation of autologous T-cells

**Fig. 2:**
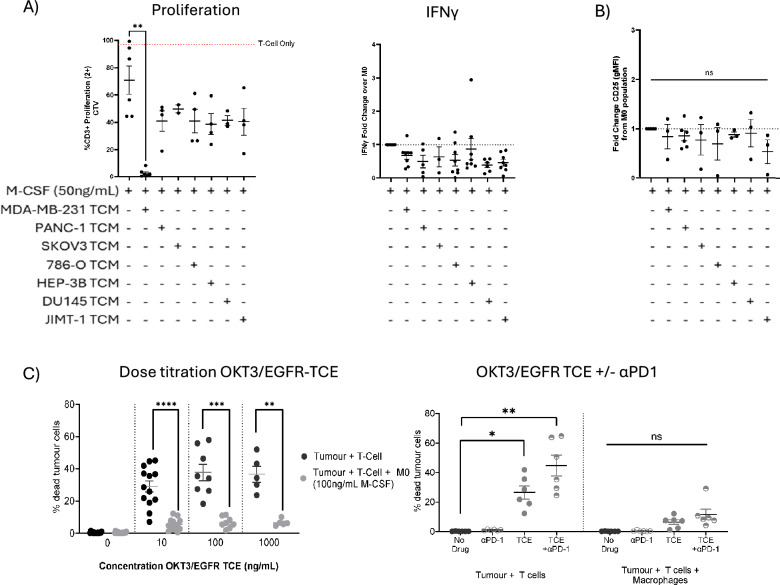
Effects of TCM-exposed hMDMs on T cells. All monocytes were isolated from healthy donor PBMCs and differentiated with M-CSF for 7 days with +/− TCM (1:1 TCM:RPMI) as indicated in the figure below before analysis. Autologous T-Cell and Macrophages used. (**A**) Flow cytometry assessment of % proliferated donor-matched CD3 + T-cells 4 days post-CD3/CD28 stimulation and co-culture with TCM-hMDMs (macrophage:T cell = 1:2). Matched IFNγ release from supernatants. (**B**) Effect of TCM-polarised hMDMs on late T cell activation marker CD25. (**C**) Flow cytometry assessment of the effect of tumour-polarised M0s on matched T cell cytotoxicity in 3-way assay in the presence of OKT3/EGFR TCE alone and in combination with 50 nM αPD1. (tumour cell: monocyte: T cell = 1.5:3:5). Cells co-cultured for 3 days before flow cytometry readout. Each point represents one donor, values shown as Mean +/− SEM. Statistical significance calculated by one-way ANOVA with Dunnet’s (**A**, **B**) or Wilcoxon matched-pairs signed rank tests (**C**) and shown where *p-value < 0.05, **p-value < 0.005 and ***p-value < 0.0005 and ****p-value < 0.0001.

To assess if the different hMDM polarisation conditions induced a functionally suppressive phenotype, we co-cultured autologous T-cells with polarised macrophages. We measured T-cell proliferation in the presence of CD3/CD28 stimulation. In this assay, M0 macrophages showed around 50% suppression of T-cell proliferation compared to T-cells alone, with some donor variability (Fig. 2A). Notably, T-cell suppression was significantly increased upon exposure to MDA-MB-231 TCM-educated macrophages (*p* = 0.0057). There was suppression observed in the presence of all other TCM, but this was not significantly different from M0 macrophages. HEP-3B, JIMT-1 and 786-O TCM also trended towards enhanced suppression, but this did not reach statistical significance due to high donor variability. In a separate experiment, IL-4/IL-13-polarised macrophages (*p* = 0.0045) or MDA-MB-231 TCM (*p* = 0.0089) showed significant augmentation of T-cell suppression (Fig. S2B).

Next, we assessed IFN-γ secretion in the media of autologous co-cultures as a measure of T-cell activation (Fig. 2A). Except for IL-4/IL-13-polarised macrophages (*p* = 0.0397 Fig. S2B), none of the TCM differentiated macrophages increased IFN-γ secretion above M0 macrophages, suggesting that T-cell activation is dampened upon exposure to TCM. We also investigated levels of the T-cell activation marker CD25 and observed no upregulation for CD25 (Fig. 2B). We also measured the early activation marker CD69, which showed an increase in CD69 expression on T cells co-cultured with TAMs polarised in MDA-MB-231 and JIMT-1 TCM (Fig. S2C). It is important to note that measuring CD69 does not always indicate a strong, sustained activation; rather it is a transient response to activation signals, including the weak or partial ones provided by TAMs and, as such CD25 expression provides a more robust measurement of sustained T cell activation in immunological studies.

In addition to T-cell proliferation, we also evaluated the ability of these cells to kill tumours. We set up a 3-way co-culture of T-cells, macrophages, and MDA-MB-231 tumour cells expressing EGFR, then exposed the co-culture to a T-cell engager (TCE) targeting EGFR (Fig. 2C). In the absence of macrophages, T-cells stimulated with various concentrations of EGFR-targeted TCE showed robust killing of MDA-MB-231 cells. Matched donor macrophages were polarised from monocytes in 100nG/mL MCSF for 7 days. When the macrophages were added to this co-culture, the ability of T-cells to kill target cells strongly declined at all concentrations of the drug as measured by flow cytometry (1000 nM *p* = 0.0026, 100 nM *p* = 0.0001 & 10 nM *p* < 0.0001). TCE-mediated killing moderately improved in the presence of anti-PD1 blockade without macrophages (although this was non-significant). This benefit, however, was abrogated in the presence of macrophages, whereby anti-PD1 blockade failed to further improve T-cell killing by EGFR TCE (Fig. 2C). The representative gating strategy for live/dead analysis is shown in Fig. S1.

### Proteomic analysis of hMDMs exposed to tumour-conditioned media

Because the use of flow cytometry to measure surface marker expression alone is insufficient to fully capture macrophage polarisation, we next examined protein expression profiles in hMDMs polarised with TCM from cancer cells^18^. Macrophage lysates from three donors were digested and analysed using data-independent acquisition (DIA) proteomic workflows. Nearly 6500 proteins were identified per sample with inter-donor variability < 20% (Fig. S4Ai and Aii).

**Fig. 3:**
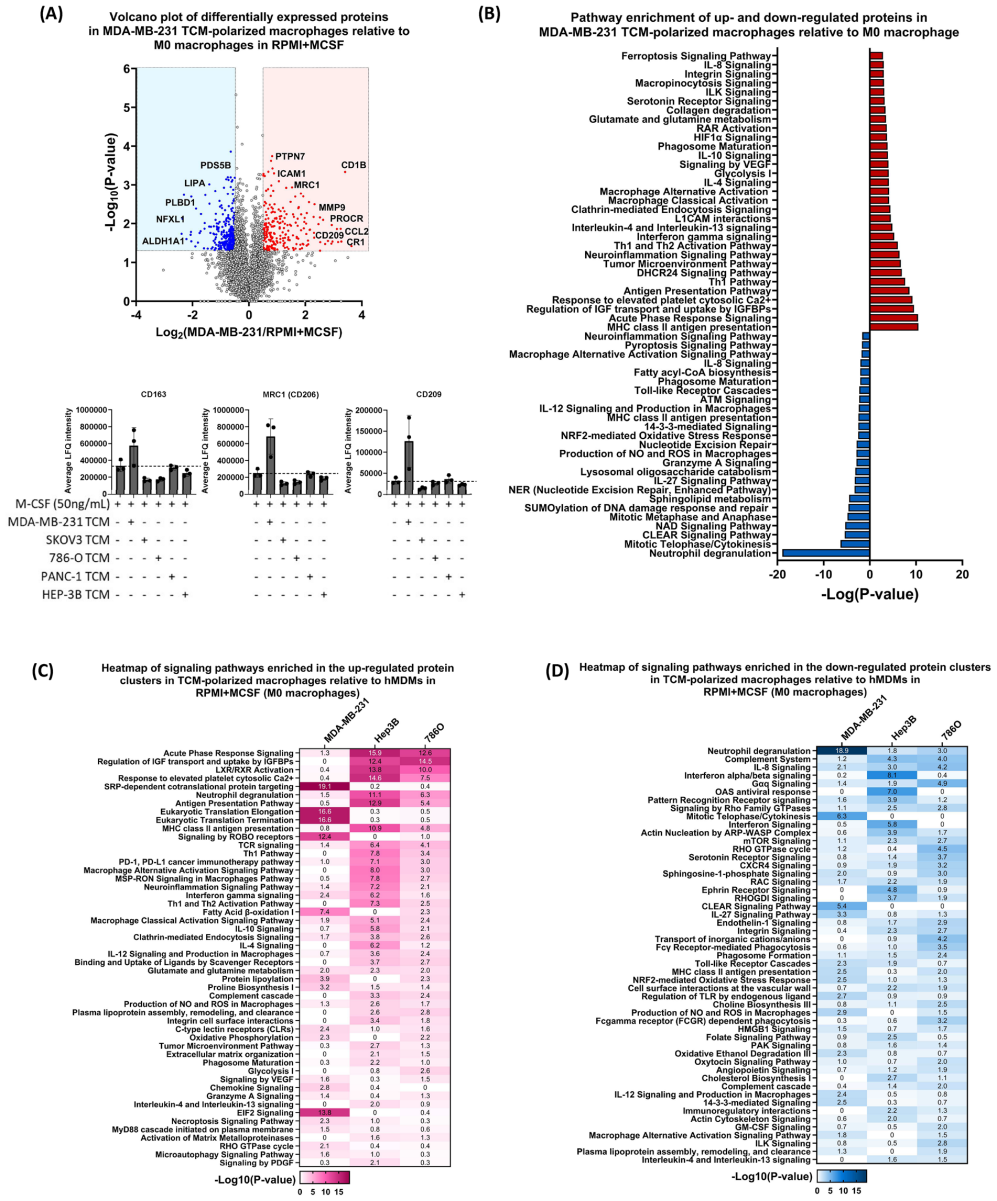
Proteomics analysis of hMDMs exposed to TCMs. All monocytes were isolated from healthy donor PBMCs and differentiated with 50 nG/mL M-CSF for 7 days +/− TCM (1:1 TCM:RPMI) as indicated in the figure below before analysis. Cell pellets were lysed via ultrasonication prior to subsequent downstream processing. (**A**) Volcano plot representing differentially expressed proteins in MDA-MB-231 TCM-exposed macrophages relative to ‘M0’ M-CSF macrophages derived using Welch’s t-test (FDR ≤ 0.05, fold change cut-off ± 0.5. Proteins highlighted in red were significantly increased and those in blue were significantly decreased in MDA-MB-231 TCM-polarised macrophages M0 Macrophages. LFQ intensities of macrophage markers previously assessed by flow cytometry– MRC1 and CD163. (**B**) Pathway enrichment of up- and down-regulated proteins in MDA-MB-231 TCM-polarised macrophages relative to M0 macrophages. (**C**, **D**) Comparative analysis of signaling pathways enriched among (**C**) upregulated and (**D**) downregulated protein clusters identified in TCM-polarised macrophages relative to M0 (M-CSF) macrophages.

Proteomic analysis showed that macrophages polarised with MDA-MB-231 TCM had numerous differentially expressed proteins compared to M0s. Figure 3A highlights these differences, including proteins like CCL2, FBP1, and MMP9, typically upregulated in suppressive macrophages. Conversely, ALDH1A1 and LIPA, usually upregulated in classically activated macrophages, were downregulated in MDA-MB-231-polarised macrophages.

Protein abundances measured by proteomics (LFQ intensities) of MRC1 (CD206) and CD163 aligned with flow cytometry findings in Fig. 1, alongside CD209, another TAM marker upregulated in MDA-MB-231 TCM-polarised macrophages (Fig. 3A).

Pathway analysis of top differentially expressed proteins in MDA-MB-231 TCM polarised macrophages versus M0 macrophages revealed several altered pathways, indicating that these hMDMs resemble suppressive TAMs. Figure 3B highlights pertinent pathways, including VEGF signalling, IL-10, IL-4, and IL-13 signalling, MHC class-II presentation, and production of NO and ROS in macrophages. The same analysis was conducted with other TCM from 786 to 0, Hep3B, SKOV3, and PANC-1 cells (Fig. S4B, C).

To systematically determine differences between macrophages polarised with suppressive cell lines (MDA-MB-231, Hep3B, and 786-O), we conducted a comparative analysis using the IPA tool relative to M0 macrophages (Fig. 3C, D). Proteins identified with increased expression in TCM-polarised macrophages were enriched in pathways like IL-4 and IL-13 signalling, glucose metabolism, VEGF signalling, and IL-10 signalling. Hep3B TCM showed the most significantly activated LXR/RXR activation, acute phase response, and DHCR24 signalling pathway, involved in stress response and cholesterol synthesis. Proteins with reduced expression in the TCM-polarised macrophages were linked to neutrophil degranulation, interferon signalling and TLR mediated pathways.

### Secretome analysis of tumour-conditioned media

The secreted factors in TCM were profiled using mass spectrometry. Approximately 5000 proteins were identified per sample, with around 30% detected in the control RPMI medium. Contaminant proteins from bovine serum were excluded from the downstream comparative analysis steps (Fig. S5A, B).

**Fig. 4:**
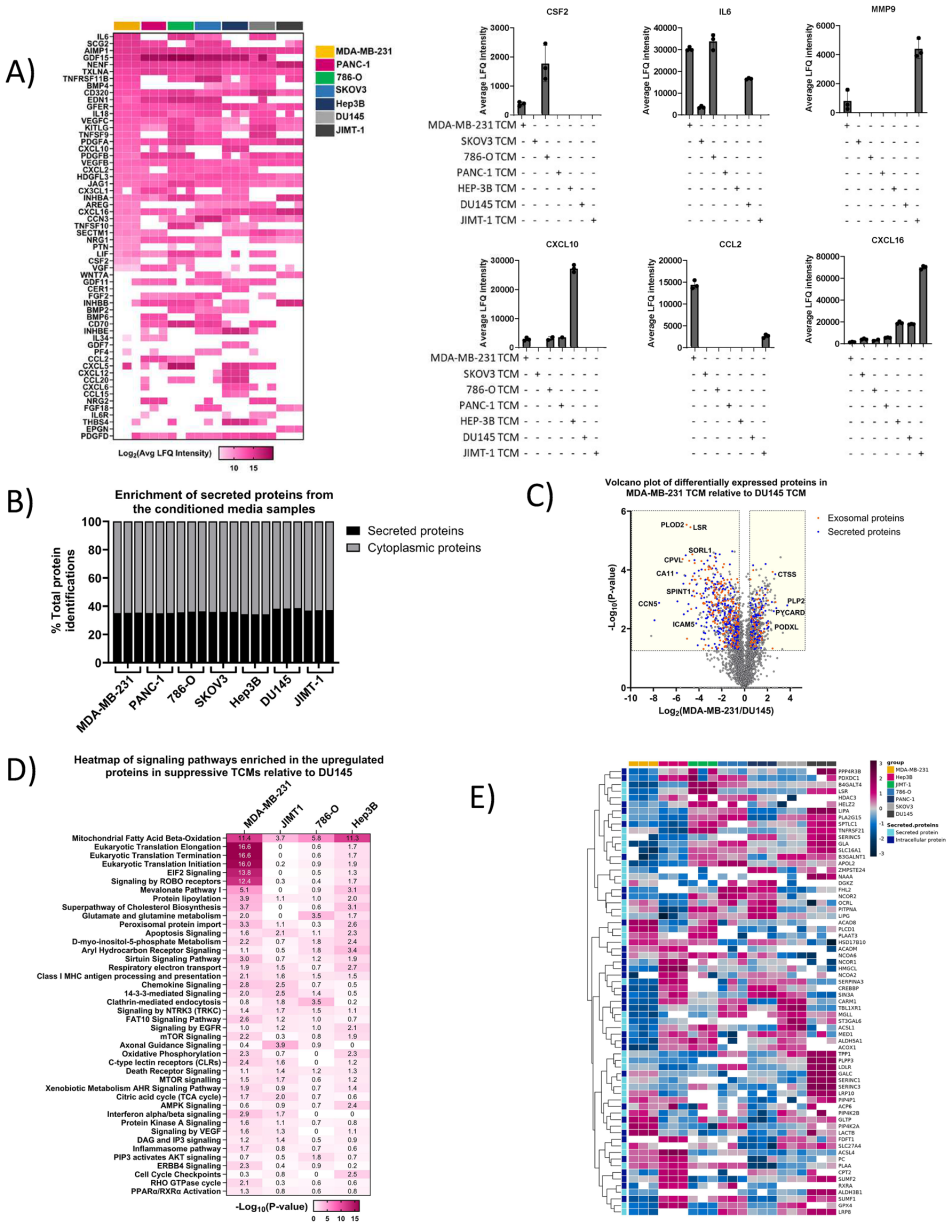
Conditioned media proteomic profiling. (**A**) Heatmap displaying Log_2_ transformed LFQ intensities of all cytokines and growth factors identified in the dataset. Bar plots showing LFQ intensities of select growth factors and cytokines. Bars represent mean values from 3 biological replicates. Error bars indicate SD. (**B**) Stacked bar chart showing percent enrichment of secreted proteins based on GO annotations (**C**) Volcano plot representing differentially expressed proteins in MDA-MB-231 TCM relative to DU145 TCM derived using Welch’s t-test (FDR ≤ 0.05, fold change cut-off ± 0.5. Proteins highlighted in blue are exosomal proteins and proteins highlighted in orange are secreted proteins. (**D**) Heatmap showing significantly enriched pathways for proteins with increased expression in suppressive TCMs compared to non-suppressive DU145 (*p*-value cutoff ≤ 0.05). (**E**) Hierarchical clustering of lipid metabolism proteins identified across all TCM samples.

In-depth evaluation of cytokines, growth factors, and chemokines present in the TCM revealed an increased expressed of proteins driving macrophage polarisation towards a TAM-like phenotype. Notably, IL-6, CSF2, and MMP9^18–20^, were found to have the highest expression in TCM from MDA-MB-231, and 786-O cells. On the other hand, proteins promoting classically activated macrophage-like polarisation, such as CXCL16, CXCL10, and CCL2^21–24^, were below the limit of detection or at relatively low abundance in suppressive MDA-MB-231 TCM (Fig. 4A).

Using Gene Ontology (GO) terms for cellular component (CC) such as “cell periphery”, “extracellular region”, “extracellular space” and Uniprot keyword “secreted”, we curated a database of 8533 unique protein IDs annotated as secreted proteins. Our analysis revealed that nearly 40% of the identified proteins in the TCM samples were annotated as secreted (Fig. 4B). The differentially regulated proteins in the TCM samples were evaluated using the non-suppressive DU145 as the baseline reference. As shown in Fig. 4C, 490 proteins exhibited increased expression, while 759 showed reduced expression in MDA-MB-231 relative to DU145, with a fold change cut-off set to 0.5 at *p*-value ≤ 0.05. GO analysis revealed many exosomal proteins among the differentially expressed proteins. Similar analyses were conducted for other TCM compared to DU145 (Fig. S5C).

Pathway enrichment analysis of proteins with increased abundance in the tumor-conditioned media (TCM) from MDA-MB-231, Hep3B, 786-O, and JIMT-1 cell lines was visualized as a heatmap (Fig. 4D). This analysis revealed that MDA-MB-231 TCM exhibited the greatest enrichment in pathways such as VEGF signaling, C-type lectin receptors (e.g., CD206 & CD209), pyroptosis, and various lipid metabolism pathways compared to JIMT-1, 786-O, and Hep3B. In a separate analysis, pathway enrichment of proteins annotated as “secreted” also showed that MDA-MB-231 was most significantly enriched in the aforementioned pathways (Fig. S5D).

Given the reported role of lipid metabolism in macrophage polarisation^19,20^, and the observed increase in lipid metabolism proteins in MDA-MB-231 TCM, we performed hierarchical clustering of all proteins involved in lipid metabolism processes. As illustrated in Fig. 4E, very few proteins exhibited consistent expression across all TCMs. Notably, proteins such as ACAD8, PC, ACADM, and HSD17B10 displayed similar expression patterns between Hep3B and MDA-MB-231. Specifically, in MDA-MB-231, PLCD1, which has been reported to suppress inflammatory responses in macrophages^21^ was found to have the highest expression.

Additionally, a cluster of approximately 30 proteins was most highly expressed in Hep3B. Most of these proteins, including CPT1A, CREBBP, and ACSL1, are involved in metabolic processes that regulate fatty acid oxidation, triglyceride metabolism, and lipid transport. Similarly, DU145 displayed high expression of a specific cluster of lipid metabolism proteins, such as GALC, PIP4K2B, and LDLR, which are essential for controlling lipid catabolic processes and transport.

### Functional validation of secretomics data

Secretomics analysis of TCM highlighted several unique factors in MDA-MB-231 TCM, including no alterations in lipid metabolism and exosomal protein abundance. To test if these factors contribute to the macrophage polarisation, we polarised hMDMs either with MDA-MB-231 TCM that had been depleted of lipids, or with TCM from MDA-MB-231 cells pre-treated with the exosome inhibitor GW4869.

**Fig. 5:**
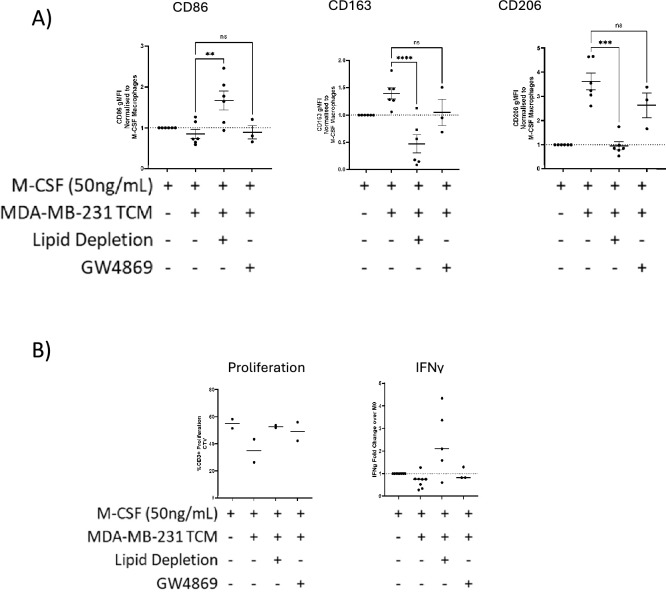
The role of lipid and exosome pathways in macrophage suppression. All monocytes were isolated from healthy donor PBMCs and differentiated with M-CSF for 7 days with M-CSF +/− TCM (1:1 TCM:RPMI) as indicated in the figure below before analysis. Autologous T-Cell and Macrophages were co-cultured for 4 days. TCMs were depleted of exosomes or lipids prior to polarisation. (**A**) CD86, CD163 and CD206 expression assessed by flow cytometry on hMDMs exposed throughout differentiation to TCM subjected to lipid depletion or exosome inhibition using GW4869. Normalised with respect to M0. (**B**) Effect of TCM lipid depletion or exosome inhibition on hMDM ability to suppress T cell proliferation (macrophage:T cell = 1:2) and IFNγ secretion following CD3/CD28 stimulation. Each point represents one donor, values shown as mean +/− SEM. Statistical significance calculated by one-way ANOVA with Dunnet’s multiple comparisons and shown where *p-value < 0.05, **p-value < 0.005 and ***p-value < 0.0005 and ****p-value < 0.0001.

We analysed the expression of CD86, CD163 and CD206 (Fig. 5A). We noted that hMDMs polarised in lipid-depleted MDA-MB-231 TCM displayed significant up-regulation of the canonical M1 marker CD86 compared to unmodified TCM (*p* < 0.0001). TAM markers CD163 and CD206 were notably reduced in hMDMs polarised with MDA-MB-231 TCM depleted of lipids (*p* < 0.0001 & *p* = 0.0015 respectively), whilst exosome depletion did not significantly affect expression.

To establish if lipid or exosome inhibition in TCM affected the ability of macrophages to suppress T-cell proliferation, we analysed T-cell proliferation. In a preliminary study, macrophages generated in MDA-MB-231 TCM with lipids or exosomes depleted were notably less suppressive than those generated in untreated TCM (Fig. 5B). Although non-significant, lipid depletion but not exosome inhibition also trended to show restored IFN-γ production from T-cells in suppressive co-cultures from some donors (Fig. 5B).

We then investigated the metabolic profile of macrophages exposed to modified MDA-MB-231 TCM using Fatty Acid Oxidation (FAO) Flux analysis following the addition of the substrate palmitate (a long-chain saturated fatty acid that serves as a substrate for FAO). Our analysis showed that whilst there was an increase in ECAR and OCR values, upon the addition of etomoxir (an inhibitor or carnitine palmityl transferase 1), ECAR and OCR values for each condition remained s across all three donors assessed (Fig. S6A). We also observed that macrophages differentiated in lipid-depleted TCM, despite showing good viability, were not sufficiently metabolically active to read out in the assay (Fig. S6B). This is to be expected as starvation triggers a cascade of metabolic changes to adapt to nutrient scarcity, including reduced respiration, shifts in fuel source preferences, and activation of processes like autophagy. This finding highlights the importance of tumour cell-derived fatty acids upon macrophage polarisation.

### Extension of findings to ascites fluid-polarised hMDMs

Ascites is the accumulation of fluid within the peritoneal cavity, occurring frequently in patients with advanced ovarian cancer (Table S2). We sought to compare the most reproducibly suppressive macrophages from our screen of TCM (MDA-MB-231 TCM) to a 50% ascites fluid polarisation condition.

**Fig. 6:**
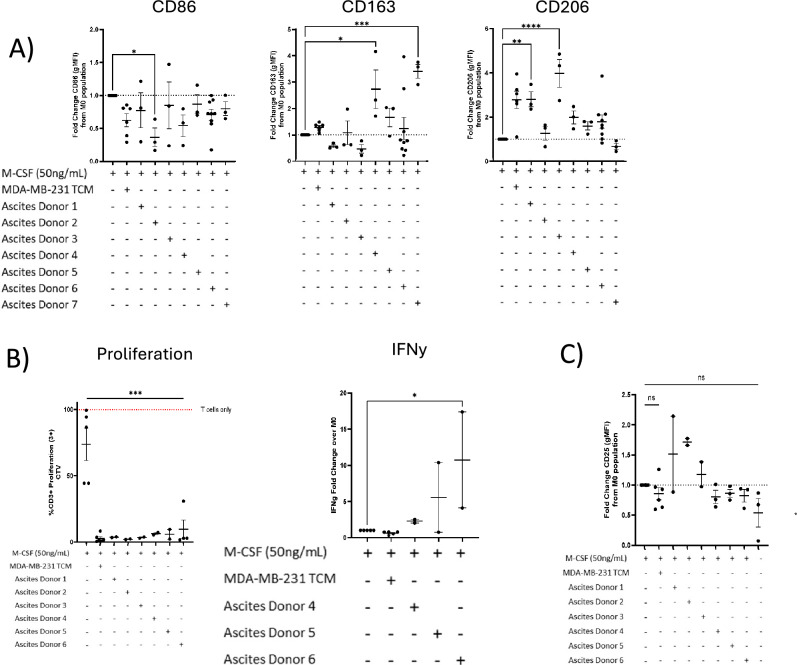
Functional and phenotypic characterization of hMDMs exposed to ascites fluid from cancer patients. All monocytes were isolated from healthy donor PBMCs and differentiated with M-CSF for 7 days with M-CSF +/− Ascites Fluid 1:1 Ascites:RPMI) as indicated in the figure below before analysis. Autologous T-Cell and Macrophages were co-cultured for 4 days. (**A**) Effect on CD86, CD163 and CD206 expression on hMDMs following ascites exposure throughout differentiation. Normalised with respect to M0. (**B**) % proliferated CD3 + T-cells and matched IFNy release from supernatants following CD3/CD28 stimulation and co-culture with ascites exposed macrophages. (**C**) Effect of ascites-polarised hMDMs on late T cell activation marker CD25. Each point represents one donor, values shown as mean +/− SEM. Statistical significance calculated by one-way ANOVA with Dunnet’s multiple comparisons and shown where *p-value < 0.05, **p-value < 0.005 and ***p-value < 0.0005 and ****p-value < 0.0001.

We analysed the expression of typical immunosuppressive TAM-markers: CD206 & CD163, and the classically activated macrophage marker CD86 on ascites fluid-polarised hMDMs. The responses were heterogenous. 2 of 7 ascites donors tested significantly increased the levels of CD206 compared to M0 macrophages (Fig. 6A). CD163 expression was variable across donors, with ascites donors 4 and 7 showing higher levels of CD163 compared to M0 macrophages. 1 out of 7 ascites donors also showed significantly decreased levels of the M1 marker CD86 compared to M0 polarised macrophages (Fig. 6A).

To determine if macrophages polarised in ascites fluids have immunosuppressive properties, we analysed autologous T-cell proliferation in ascites-pre-exposed macrophage co-cultures. Remarkably, all ascites fluid-polarised hMDMs induced significant blockade of T-cell proliferation (Fig. 6B). We also analysed IFN-γ secretion in the co-culture media as a biomarker of T-cell activation. Although IFN-γ release was generally low, two of the ascites donors (donors 5 & 6) showed elevated IFN-γ expression, with high variability among donors (Fig. 6B). In a similar pattern to hMDMs polarised with TCM, ascites fluid polarisation did not result in the expression of CD25 on T-cells (Fig. 6C). This demonstrates that TCM and primary ascites fluid polarisation can induce similar immunosuppressive phenotypes in macrophages based on classical marker expression and inhibition of T-cell functionality. One key difference was that ascites fluid was more efficient at blocking T-cell proliferation than most TCM evaluated in our screen.

### Transcriptomics analysis comparing primary human cancer datasets to hMDMs polarised by ascites fluid and tumour-conditioned media

**Fig. 7:**
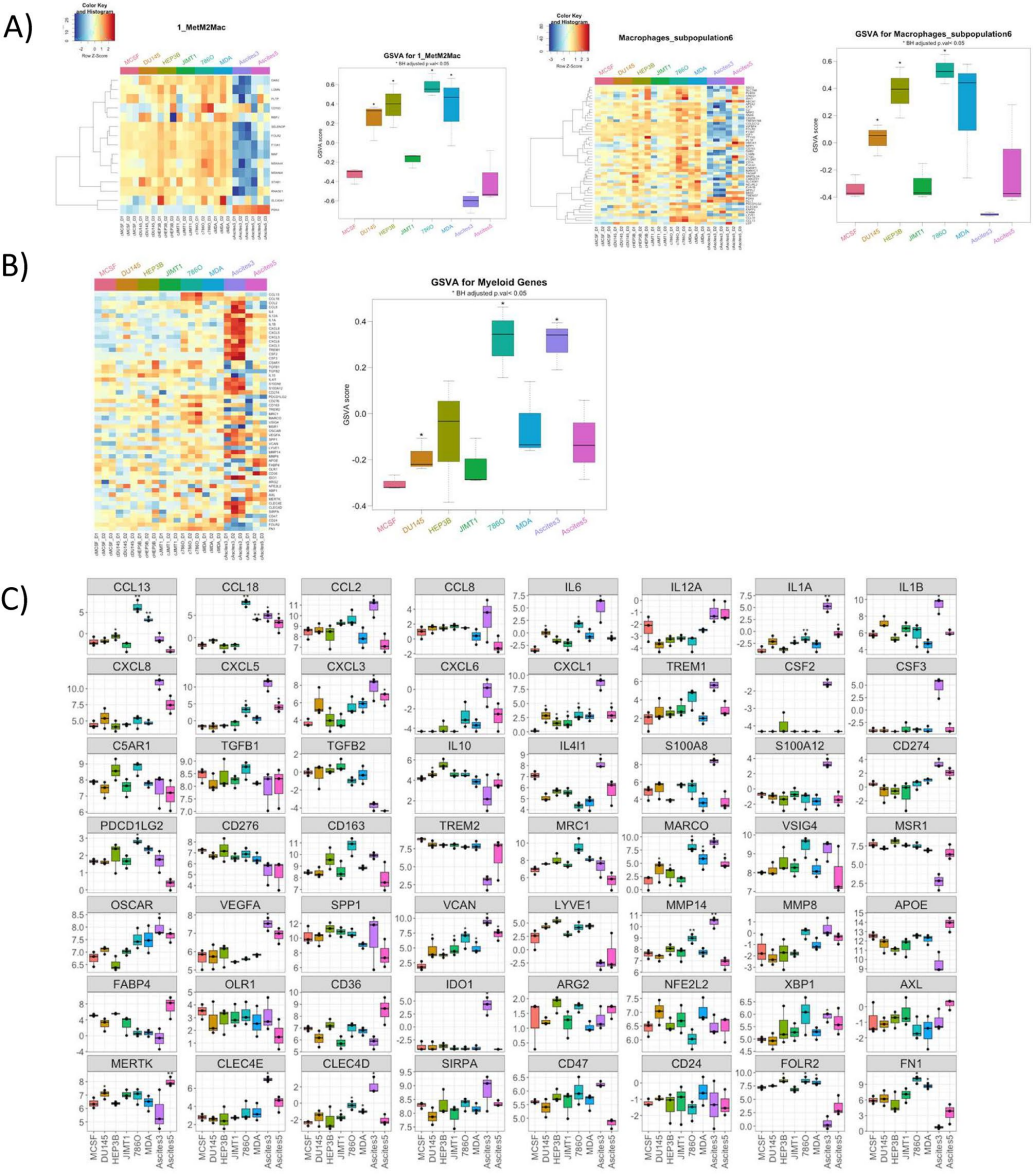
Transcriptomics analysis of hMDMs exposed to TCMs throughout differentiation. All monocytes were isolated from healthy donor PBMCs and differentiated with 50 nG/mL M-CSF for 7 days +/− TCM (1:1 TCM:RPMI) as indicated in the figure below before analysis. Cell pellets were frozen and shipped to Azenta Genewiz for sequencing. (**A**) Heatmaps of scaled log2-transformed gene expression data across conditions from published TAM signatures. Boxplots of GSVA scores per sample of published TAM signatures. (**B**) Gene expression heatmap and GSVA scores boxplot as in A for a selected list of myeloid relevant genes. (**C**) Boxplots of gene expression for a selected list of myeloid relevant genes. *Benjamini-Hochberg (BH) corrected *p*-values < 0.05 compared to monocytes polarised in RPMI + 50nG/mL M-CSF.

To confirm our findings, we used RNA Sequencing analysis to gain insight into the transcriptomic fingerprint of in vitro differentiated macrophages, and their relation to primary human cancer datasets by performing gene set variation analysis (GSVA) with published TAM signatures. Based on this analysis, MDA-MB-231-TCM-polarised macrophages showed significantly higher GSVA scores for a specific TAM subtype characterised by FOLR2, SELENOP, and CD163 expression (MetM2mac, Fig. 7A)^22^ indicating a high similarity of these macrophages to these primary human TAM populations. These MDA-MB-231 also showed a high similarity to a separate primary dataset characterised by the expression of CCL18, FOLR2 and LYVE1 (subpopulation 6)^23^ although this did not reach significance. Half of the genes in the MetM2mac signature overlap with the subpopulation 6 genes, suggesting they may be capturing similar macrophage cell states. These data show that MDA-MB-231-TCM polarised macrophages show similarity to populations of TAMs found in patients. Likewise, 786-O, HEP3B and DU145 TCM-polarised macrophages also showed significantly higher GSVA scores for these two populations (Fig. 7A).

Both ascites samples selected for further analysis showed significantly higher GSVA scores for a distinct macrophage subtype (subpopulation1, Fig. S7A)^23^, while ascites 5 and 786-O TCM-polarised macrophages induced features of another macrophage population (subpopulation 3, Fig. S7A)^23^. Notably, comparison of monocytes polarised with IL-4/IL-13 cytokines (a commonly used polarisation protocol for modelling suppressive TAMs in vitro) had much lower GSVA scores for both subpopulation 6 and MetM2Mac compared to macrophages polarised in MDA-MB-231 TCM (Fig. S7B). This finding highlights that TCM-polarised macrophages represent a more clinically relevant TAM model compared to the widely used IL-4/IL-13 model of TAM polarisation.

Next, we performed a more supervised analysis approach, curating a list of myeloid genes associated with pro-inflammatory mediators, differentiation drivers, suppressive mediators and markers, myeloid checkpoints, angiogenesis & tissue repair, extracellular matrix remodelling, metabolism & lipid procession, oxidative stress response, and efferocytosis. We then assessed GVSA scores for this custom gene signature, and 786-O- and ascites 3-polarised macrophages showed a strong enrichment for this signature (Fig. 7B).

Consistent with our pathway analysis, monocytes polarised in MDA-MB-231 TCM showed significant upregulation of genes typically associated with immunosuppressive alternatively activated macrophages, such as chemokines CCL13, CCL18, the myeloid checkpoint MARCO, as well as genes involved in angiogenesis, matrix reprogramming and efferocytosis (VCAN, MMP14, and CLEC4D, respectively) as well as CXCL1 which is associated with classically activated macrophages (Fig. 7C). Monocytes polarised in 786-O TCM showed a similar profile to MDA-Mb-231-TCM-exposed cells, also upregulating genes like CCL13, CCL18, PDCD1LG2 (PD-L2), MARCO, VCAN, and CLEC4D,

Ascites donor 3-polarised macrophages stood out from TCM conditions in their ability to upregulate genes associated with angiogenesis, such as VEGFA and VCAN, and also showed strong enrichment of MMP14, IDO1, S100A8, S100A12 genes and efferocytosis genes CLEC4E and CLEC4D. Macrophages polarized with this condition also upregulated myeloid checkpoints OSCAR and MARCO. Intriguingly, despite this profile, ascites donor 3 polarisation induced high expression of pro-inflammatory genes, such as IL6, IL1A, IL1B, CXCL8, CXCL5, CXCL3, CXCL1 (Fig. 7C). Notably, this pattern implies a hybrid phenotype, which is often observed in complex in vivo environments, further suggesting that this polarisation tactic can produce a more physiological TAM-like phenotype^24–28^.

Finally, Ascites donor 5-polarized macrophages were strong expressors of MERTK, which was distinct from ascites donor 3. They also showed a trend toward upregulation of lipid mediators APOE, FABP4 and CD36 (Fig. 7C). Interestingly, we observed opposite trends in the two ascites samples tested, with ascites 3 and ascites 5 being enriched and suppressed, respectively, in inflammatory pathways related to IFNγ (Fig. S7C).

Overall, our results indicate that there is no one-size-fits-all in vitro model for TAMs. However, exposing macrophages to TCM from cell lines such as MDA-MB-231 and 786-0, and primary ascites fluid can induce translationally relevant populations of macrophages that capture specific aspects of TAM suppressive phenotypes seen in patients, be it angiogenesis, extracellular matrix remodelling, myeloid checkpoint expression, or inflammation. It is notable that certain polarization conditions showed strong bias for upregulation of specific TAM markers such as MARCO, MERTK, IDO1, IL6, CCL2, IL1A/1B and S100A8/A9. This, along with the ability of polarising conditions (such as MDA-MB-231 TCM and ascites fluids) to produce TAMs that reliably suppress T cell proliferation should be factored into the choice of a suitable and clinically relevant model to study specific aspects of human TAM biology in vitro.

## Discussion

The binary classification of macrophages into M1 and M2 states oversimplifies the complex spectrum of macrophage polarisation^29^ Instead, macrophage polarisation represents a continuum, with M1 and M2 as its polar extremes. This spectrum, along with the plasticity of macrophages, makes studying human macrophages in vitro challenging. Traditional methods for studying TAMs involve polarising hMDMs using M-CSF and high levels of recombinant cytokines like IL-4 and IL-13. While these approaches can create macrophages with suppressive properties and increase immunosuppressive TAM markers such as CD163 and CD206, they fail to adequately reproduce the complex TAM phenotypes found in vivo. This discrepancy is crucial given the rate of clinical failure of immunotherapy drugs which in part can be attributed to the poor predictive power of current pre-clinical models. Thus, there is an urgent need to develop more translationally relevant in vitro models of human TAMs^30–34^.

To address these challenges, our research used TCM or ascites fluids from cancer patients to mimic the diverse factors that macrophages encounter in the TME. We analysed macrophage polarisation states induced by these conditions across a broad range of assays, with the dual aim of identifying reliable, high-throughput models of TAM-like cells as well as gaining deeper understanding of the translational relevance, and limitation of each model. TCM models have been previously described in the literature as providing a novel method to study macrophages in vitro, and we aimed to compare such protocols to clinical samples to understand their translational relevance^35^.

While our models do not fully capture cell–cell interactions affecting TAM polarisation, they effectively induce pro-tumoural phenotypes observed in TAMs. Specifically, we found that TCM from MDA-MB-231 breast cancer cells and ascites fluids were the most robust methods to generate consistently suppressive TAM-like populations, suitable for high-throughput drug screening and functional analysis. These can be particularly useful for screening novel macrophage directed therapies as an initial de-risking strategy. For much of the downstream analysis, our research focused primarily upon TCM from MDA-MB-231 cells for these reasons. Furthermore, although other TCM, such as 786-O and HEP3B, induced suppressive TAM-like cells with higher donor-to-donor variability, our transcriptomics analysis showed they aligned well with TAM signatures in cancer patients, so can be applicable and add value in less high-throughput settings.

Our findings reiterate that no one universal in vitro model captures the complexity of human TAMs. Instead, a panel of validated polarising conditions is more suitable for representing specific TAM-like phenotypes and functionalities, reflecting heterogeneity seen in patients. We here identify at least 3 different TCM and ascites fluid conditions that align with specific TAM population signatures in patients and together can recapitulate most of TAM features, including angiogenesis, myeloid checkpoint expression, proinflammation, efferocytosis, and matrix remodelling. For example, we show that ascites donor 3 conditioning is suitable for modelling pro-inflammatory and angiogenic TAM populations, ascites donor 5—MERTK-enriched TAM populations and 786-O TCM—PD-L2- and MARCO-enriched TAM populations. Future in-depth proteomic analyses of patient-derived ascites fluid may enable the identification of reproducible biomarkers and provide mechanistic insights into the drivers of macrophage reprogramming. Most of these signatures were notably not replicated by polarisation with recombinant IL-4/IL-13 cytokines, which is commonly used to model TAMs in vitro. Importantly, we demonstrate that TCM and ascites fluid polarisation of macrophages is more translationally relevant through alignment to primary TAM signatures derived from primary human cancer datasets.

We show that ascites donor 3 conditioning induces a hybrid macrophage phenotype characterized by upregulation of both inflammatory and suppressive genes. The concept of a hybrid macrophage state (namely macrophages which express both pro-inflammatory and anti-inflammatory markers and functions) does not fit the classical M1/M2 paradigm. This classification has gained significant attention in recent years, with several papers suggesting it is a more reflective of TAM populations observed in vivo^24–28^. 786-O TCM-polarised macrophages also showed this pattern and had significantly enriched GSVA scores for several primary TAM signatures even though they were less able to reliably suppress T cell proliferation in in vitro co-cultures. MDA-MB-231 TCM-polarised macrophages, on the other hand, showed better alignment to TAM cluster characterised by FOLR2, SELENOP and CD163 expression (MetM2mac) and demonstrated significant upregulation of several TAM-associated genes such as MARCO, FOLR2 and CLEC4D; this, along with their ability to consistently suppress T cell proliferation across donors makes them a suitable workhorse model for many in vitro assays. Macrophages polarised with MDA-MB-231 TCM, however, do not display significant upregulation of genes such as IDO1 and MMP14, re-iterating the fact that there is no single universal model for studying all aspects of TAM biology in vitro.

Of note, PANC1 cells have previously been found to generate suppressive environments for macrophage polarisation, yet this has not induced a suppressive phenotype in our assay^36^. This can partly be explained by differing methodologies, as the authors generated TAMs without M-CSF, and diluted TCM to 30% with a polarisation length of 5 days. Likewise, we have not studied cell–cell contact co-cultures but rather the effect of TCM alone on polarisation, however, further work is needed to verify if this explains this discrepancy.

Although CD163 and CD206 have been defined as immunosuppressive TAM markers, often used alongside markers like CD86 and MHC-II to differentiate between classically activated and suppressive TAMs^37^, we have shown that their expression levels do not directly correlate with the suppressive ability of TCM-differentiated macrophages in vitro. Specifically, we observed up-regulation of CD206 and CD163 by flow cytometry, proteomics, and RNAseq in macrophages polarised with suppressive MDA-MB-231 TCM, 786-O TCM, and ascites fluids. However, other TCM, such as HEP3B, which also aligned with patient TAM signatures, did not upregulate these markers, questioning the validity of CD206 and CD163 as reliable biomarkers of suppressive TAMs.

The interaction between T-cells and the TME, particularly TAMs, can shape the immune response to cancer, so it is important to model in vitro to assess the success of T-cell-targeting therapies such as PDx. As seen in our assessments of T-cell suppression paired with IFNγ secretion, despite some of the TCM-polarised macrophages being suppressive, the production of IFN-γ by T-cells remained largely unaffected, perhaps due to co-stimulatory signals primarily driving IFN-γ production. It is possible that the use of potent T-cell activators like recombinant CD3/CD28 overrides the ability of TAMs to inhibit IFN-γ release once T-cells have been activated. Exploration of alternative, more physiological activation conditions, e.g. using antigen stimulation, is warranted to explore this phenomenon further. We extended our findings by assessing the role of macrophages polarised in the presence of MDA-MB-231 cells in a three-way PDx resistance T-cell killing assay we developed, which could serve as a tool for predicting and studying treatment failure. We demonstrated the inability of PDx inhibition to reverse MDA-MB-231-mediated macrophage suppression in these settings, which suggests a potential tumour immune evasion strategy and underscores the need for novel therapeutic strategies aiming to modulate macrophage polarisation states. These data highlight changes in macrophage polarisation states, which can in turn affect T cell activity, and does not identify the macrophage-dependent mechanisms responsible for T cell inhibition. Given that we find our model of macrophage polarisation to a be clinically relevant model, we suggest that this could serve as a platform for deeper mechanistic investigations into macrophage suppression of T cell function moving forward.

While classically activated macrophages and alternatively activated macrophages have well established contrasting phenotypes, we have shown that TCM can polarise macrophages to a unique metabolic profile, distinct from those differentiated with M-CSF. Classically activated macrophages are observed to be glycolytic with increased FAS and reduced mitochondrial metabolism^38^. These macrophages accumulate itaconate and succinate due to alterations in the TCA cycle, which in turn inhibits succinate dehydrogenase, causing HIFα stabilisation and the onset of glycolytic gene transcription activation^39^. Alternatively activated macrophages are characterised by their reliance on mitochondrial metabolism, such as FAO and oxidative phosphorylation, while having a complete TCA cycle and increased glutamine catabolism^40^.

Mito Stress Test flux analysis indicated that, despite being functionally suppressive, MDA-MB-231 and 786-O TCM-polarised-macrophages displayed higher metabolic activity than M0 macrophages. Notably, upon glucose addition, MDA-MB-231 TCM-polarised-macrophages demonstrated enhanced glycolytic capacity, complimenting prior work highlighting TAMs as the greater consumer of intratumoral glucose^41^.

Transcriptomic analysis revealed upregulation of genes related to fatty acid oxidation (FAO) and modulation of metabolic pathways by different TCM, prompting further investigation into FA metabolism. FAO Seahorse flux analysis showed differences in glycolysis, not FAO. While OCR increased with palmitate supplementation, no OCR reduction occurred following etomoxir addition, suggesting that FA pathways may not solely indicate changes to metabolic phenotype. TAMs have been shown to have metabolic plasticity, responding to nutrient depletion within the TME. Further research into these 28 mitochondrial variations is warranted. It is notable that TCM from 786 to 0 cells generated suppressive TAMs in some donors. These cells lack the von Hippel-Lindau (VHL) gene resulting in constitutive expression of HIF-2α even in normoxia, and a loss of functional HIF-1α. This accumulation of HIFs can have wide-ranging effects on the tumour microenvironment, including macrophage polarisation and highlights the importance of understanding the role of tumour cell metabolic profiles on macrophage polarisation. Although this is beyond the scope of this manuscript, we encourage further research into this area.

Considering the significant variations in the ability of different TCM to induce TAM-like phenotypes, we conducted a detailed compositional analysis of the TCM. A recent review has proposed several mechanisms by which intracellular proteins are exported into the extracellular compartment^42^. These proteins can perform extracellular functions, which can differ from their intracellular roles^42,43^. The significant presence of cytoplasmic proteins in the secreted fraction could also result from non-physiological stress or mechanical injury, leading to cellular lysis during the collection of conditioned media. It was notable that several cytokines known to drive macrophage polarisation towards a TAM-like phenotype were observed to increase in abundance in TCM generated from MDA-MB-231 cells, such as IL-34, along with CSF2 (GM-CSF) as well as CXCL12^44–46^. CSF-2 (GM-CSF) was high in MDA-MB-231 cells and 786-O, unlike any other TCM, which, while traditionally associated with the activation of macrophages, can also play a role in the differentiation of MDSCs^47,48^.

Exosomal and lipid metabolism proteins were two notable factors that were upregulated in suppressive TCM, e.g., from MDA-MB-231 cells. Inhibition of exosome generation or depletion of lipids from TCM confirmed that these factors are partly responsible for the polarisation of TAMs towards a suppressive phenotype, although this data lacked statistical significance. Other papers have noted that lipid metabolism is a key factor in this process, and that suppressive TAM phenotypes can also be established by supplementation of fatty acids during polarisation^19^. Previous reports note strikingly similar responses on macrophage polarisation with the use of cleanascite to deplete lipids from TCM^20,49,50^. Testa et al. also noted that the TCM polarisation of THP-1 cells could be blunted by adding etomoxir (an inhibitor of fatty acid metabolism)^49^. Equally, there is increasing body of evidence that exosomes can play an important role in immune education and suppression within the tumour microenvironment^51^. Peng et al. showed that exosomes derived from prostate cancer cells can skew macrophages towards a TAM-like phenotype, and that GW4869 can inhibit this process^52^. Although we have not identified which precise factors present in the TCM are responsible for the polarisation of monocytes towards a suppressive TAM phenotype, this study reemphasises the importance of such secreted factors in enforcing a suppressive microenvironment and the possibility of reversing myeloid suppression with targeted inhibitors of lipid metabolism or exosome signalling.

Secretomes derived from TCMs can contain a complex mixture of cytokines, growth factors, metabolites, and extracellular vesicles that can alter the metabolism of TAMs. For example, secretomes rich in lactate and interleukins such as IL-6 and IL-10 may enhance aerobic glycolysis in TAMs and suppress their ability to present antigens or produce inflammatory mediators^53–56^. Other components such as TGF-β and exosomal miRNAs can alter TAM metabolism through modulation of Phosphoinositide 3-kinase (PI3K)/protein kinase B (AKT) network, which can increase the expression immunosuppressive factors in the TAMs^57–59^. It has also been shown that TAMs are metabolically plastic and are able to change their metabolic phenotype in response to changes in the TME^60^. Our study reemphasises no one factor in a complex material such as TCM will be solely responsible for inducing a suppressive phenotype. Importantly, such a complex milieu cannot be replicated using recombinant cytokines to polarise healthy donor monocytes alone.

Our findings are based on a relatively narrow selection of tumour cell lines. Future research should expand the panel of tumour cell lines and employ more precise molecular tools to dissect the complex regulatory networks that govern TAM polarisation. To enhance the translational relevance of in vitro TAM models, subsequent studies could explore the use of three-dimensional models, organoids, and multicellular systems incorporating cancer-associated fibroblasts (CAFs). These advanced approaches would provide a more comprehensive understanding of TAM–CAF crosstalk and its impact on TAM polarisation and function within the tumour microenvironment (TME). The adoption of more complex models is expected to decrease experimental throughput, which may limit their suitability for initial screening of potential tumour therapies. However, such models have consistently been shown to increase translational relevance. The authors encourage future researchers to carefully consider these factors when analysing their own work.

By expanding the panel of cell lines used to generate tumour-conditioned media (TCM), it may be possible to identify common mutations that correlate with the generation of immunosuppressive TAMs. Any observed correlations should then be empirically tested for their impact on macrophage immunosuppression. Our initial analysis of the mutation profiles of the cell lines used in this study found no association between the ability to generate clinically relevant TAM models, and the presence of specific mutations; nevertheless, further investigation in this area is encouraged.

Additionally, examining the responses of different T cell subsets (e.g. CD4+ and CD8+ T cells) or analysing the production of cytolytic molecules such as granzyme B or perforin in response to co-culture with TAMs generated by our methods may enhance understanding of T cell biology and adaptive immune targets in cancer. These areas, however, fall outside the scope of this paper.

As discussed, the polarisation of healthy donor monocytes towards more clinically relevant TAM models is likely influenced, at least in part, by specific proteins and metabolites present in the TCM. The precise molecules involved, however, remain to be identified.

Our study highlights the challenges of modelling TAM in vitro and offers some solutions to modelling human TAM polarisation states in vitro using conditioning of widely accessible hMDMs from healthy donors, challenging existing paradigms of cytokine-driven polarization and highlighting the complexity of TAM biology. Our data suggest that a commonly used method of TAM polarisation (using IL-4/IL-13) is less clinically relevant than using TCM-polarisation protocols (Fig. S7B) as the crosstalk between the TME and TAMs involves multiple factors including cytokines, metabolites, and signalling pathways that are better represented in a TCM-polarisation protocol. Our data, in particular the novel comparison of these various TCMs to clinically relevant samples, places further importance upon such protocols for future research. By developing more clinically relevant in vitro models and exploring the molecular mechanisms underlying TAM polarisation, we can pave the way for successful novel therapeutic strategies targeting TAMs in cancer. Our findings also underscore the importance of considering the spectral model of macrophage polarisation and the need for a multifaceted approach to accurately validate and model TAM phenotypes.

## Methods

### Cell line maintenance

Cell lines were maintained in RPMI 1640 + glutamax + 10% Heat Inactivated Fetal Bovine Serum and 1% Penicillin/Streptomycin (10 mG/mL) (all obtained from Gibco) in a humidified 37 °C incubator (5% CO_2_). Cell lines were purchased from ATCC, routinely tested for mycoplasma, and were CST verified before use. Cells were routinely passaged when confluence rose above 80% and were discarded once the passage number exceeded 10.

### Creation of tumour-conditioned media

Once the tumour cell lines reached ~ 70% confluency, cells were incubated for 72 h in serum-low medium (RPMI + 0.1% HI FBS). After this time, the media was harvested, and cellular debris was removed by centrifugation. FBS was added to a final concentration of 10% to allow for FBS concentrations to be matched across all treatment groups. The serum-low medium was supplemented with 10 μM GW4869 (Sigma Aldrich #D1692) for exosome inhibition before incubation. For lipid depletion, tumour-conditioned media was mixed 1:2 with Cleanascite (Biotech Support Group) (and lipids were removed according to the manufacturer’s protocol.

### Isolation of monocytes from healthy donor PBMCs

Leukocyte cones were obtained from NHS Blood and Transfusion at Addenbrookes Hospital. The concentrated blood was diluted with 30 ml of PBS. Falcon tubes were prepared containing 15 ml Ficoll Paque Plus (Sigma) and 20 ml of the diluted blood was layered on a Ficoll gradient. The gradients were centrifuged at 400 G for 40 min without brakes, and the PBMC monolayer was collected. Monocytes were isolated using the EasySep Human Monocyte Enrichment Kit without CD16 depletion (Stemcell #19058). The negative fraction was collected, and monocytes were resuspended in RPMI + 10% HI FBS + Pen/Strep.

### Macrophage differentiation

All macrophages were differentiated from freshly isolated monocytes over 6 days at 37 °C, 5% CO_2_. On day 0, the monocytes were seeded at 1 × 10^6^/mL in a T175 Corning Flask in a volume of 35 mL. The media was replenished with an additional 35 ml on day 3. ‘M0’ macrophages were differentiated in the presence of 50 ng/ml M-CSF (Peprotech) supplemented at day 0 and day 3. TAMs were differentiated in a 1:1 ratio of TCM and standard media supplemented with 50 nG/mL M-CSF. For ascites-fluid polarised macrophages, ascites fluid purchased from Discovery Life Sciences, was filtered through a 22 µm filter, then diluted 1:1 with RPMI plus 50 ng/mL M-CSF then fed on day 3 with the same solution.

### Flow cytometry analysis of macrophage polarisation

For flow analysis, cells were harvested on day 6 using a non-enzymatic cell dissociation solution, stained using a NiR live/dead dye (ThermoFisher) and resuspended in flow buffer (PBS, 2% BSA, 0.1% Sodium Azide, 2 mM EDTA) supplemented with Fc block (InnovexBio #NB309). Primary labelled antibodies were used as per the supplier’s instructions. Cells were incubated in the dark at 4 °C for 30 min for all incubation steps. Cells were fixed using 3.7% PFA for 20 min at room temperature, and prior to analysis, cells were resuspended in 100 μL of cold PBS.

A BD LSRFortessaTM or BD FACS Symphony cytometer were used to measure fluorescence using DIVA software. For all flow cytometry analyses, voltages were determined using control samples, and compensation was calculated using UltraComp eBeads™ (ThermoFisher). Cellular debris was excluded through gating on the bulk population using FSC-A/SSC-A, live cells were identified by gating on the live/dead- population and single cells were identified using FSC-A/FSC-H gating. Data analysis was performed using FlowJo V10.6.1 (BD Biosciences), and the geometric mean for each fluorophore was calculated and plotted as Mean Fluorescence Intensity (MFI) using GraphPad (Prism 8).

### Flow cytometry analysis of T-cell proliferation and activation

Matched PBMCs from each leukocyte cone were frozen at − 80 °C in Cryostor CS10 cell freezing media (StemCell). Monocytes from each cone were differentiated to “M0” macrophages as detailed above (50 ng/ml M-CSF (Peprotech) supplemented at day 0 and day 3) or to TAMs through differentiation in the presence of TCM or ascites fluid as detailed above. On day 5 of the macrophage differentiation, PBMCs were revived and rested overnight in RPMI containing glutamax supplemented with 10% HI FBS + 1% Pen/Strep at 2–3 × 106 cells per mL at 37 °C. A 96-well round bottom plate was coated overnight at 4 °C in anti-CD3 (ThermoFisher #16-0037-025) diluted to 1μG/mL in PBS.

On day 6, T-cells were isolated from the PBMCs using EasySep™ Human T-cell Enrichment Kit (StemCell #19051) according to the manufacturer’s instructions, then labelled using cell trace violet (Thermo Fisher) through staining in 0.5uMol of solution for 10 min at 37 °C.

TCM-exposed, ascites-exposed and ‘M0’ Macrophages were co-cultured alongside autologous T-Cells at a Macrophage:T-cell ratio of 1:2 along with 2μG/mL anti-CD28 (ThermoFisher #16-0289-38) for 4 days, at which point supernatants were collected for further analysis. T-cells were collected and stained for flow cytometry analysis and cell trace violet T-cell proliferation analysis (Supplementary Fig. 7).

### 3-way assay

PBMCs from leukocyte cones were used to isolate monocytes and subsequently differentiated into ‘M0’ macrophages through culturing in RPMI + 10% HI FBS + 1% Pen/Strep supplemented with 100nG/mL M-CSF (Peprotech supplemented at day 0 and day 3). The remaining PBMCs were frozen at 1 × 10^8^/mL in Cryostor CS10 cell freezing media (Stemcell) and stored at − 80 °C. On day 7, the MDA-MB-231 tumour cell line and differentiated M0 macrophages were harvested and co-cultured in a 96-well U-bottom plate (15,000 MDA-MB-231 cells, 30,000 M0 macrophages). Matched PBMCs were thawed, and T-Cells were isolated using the EasySep™ Human T-cell Enrichment Kit (StemCell #19051). T-cells were resuspended in RPMI + 10% HI FBS + 1% P/S and rested overnight in culture flasks at 37 °C with 5% CO_2_. The following day, molecules and 50,000 T-cells were added to each well. Anti-PD1 monoclonal antibody (in-house) was used at 50 nM, while the OKT3-EGFR T-cell engager (in-house) was tested at 1000, 100 or 10 ng/mL. Cells were co-cultured for 3 days at 37 °C with 5% CO_2_. The proportion of dead tumour cells was assessed by flow cytometry (BD Fortessa) using live/dead fixable stain (Thermo Fisher) according to the manufacturer’s protocol (Supplementary Fig. 1).

### Ascites donors

Ovarian cancer patients undergoing routine paracentesis were identified, and informed consent was given to collect ascitic fluid in accordance with local institutional ethics review and approval. Discovery Life Sciences LLC acquired these anonymised primary human malignant ascites fluid samples. Cells were pelleted by centrifugation, and supernatants were collected and frozen for future analysis.

### Conditioned media sample preparation for proteomic analysis

Macrophages were generated as outlined above, and 1e6 cells from 3 donors and nine different conditions were collected, centrifuged, supernatants removed, and cell pellets frozen on dry ice.

For each cell line, three 800 µL aliquots of the conditioned media (CM) were processed in parallel. RPMI media containing 0.1% HI FBS served as a control to profile FBS contaminant proteins. The CM aliquots were mixed with four times their volume of ice-cold acetone and incubated overnight at − 20 °C. Samples were centrifuged for 10 min at 15,000 × *g*. The supernatant was discarded, and the resulting protein pellets were resuspended in 100 µL of EasyPep™ lysis buffer. Subsequent sample processing was carried out using the EasyPep™ Mini MS Sample Prep Kit (ThermoFisher #A40006) according to the manufacturer’s protocol. The samples were incubated overnight at 37 °C with Trypsin/Lys-C Mix, added at an enzyme/protein ratio of approximately 1:20. Peptides were desalted using peptide clean-up columns, dried under vacuum, and reconstituted in 0.15% formic acid. Peptide quantification was performed using the Micro BCA™ protein assay kit (ThermoFisher #23235), with RPMI media control samples. 200 ng of the peptide mixture was loaded on the Evotips (Evosep) per the recommended protocol.

### TAM sample preparation for proteomic analysis

Proteomic analysis was conducted on three biological replicates. Cell pellets were lysed via ultrasonication, and 25 µg protein extracts were subjected to tryptic digestion using a magnetic bead-based Solid-Phase Single Pot Sample Preparation (SP3) workflow described by Hughes et al. 2019^61^. Subsequent steps were automated using a Kingfisher Flex system (Thermo Fisher), as described previously^62^. The protein mixtures were incubated with a 1:1 mix of hydrophilic and hydrophobic carboxylate beads (Cytiva) added at a 10:1 bead/protein ratio. The bead-bound proteins were washed twice with 70% ethanol and once with 100% acetonitrile. They were then digested with Trypsin/LysC mix (Promega) added at an enzyme/protein ratio of 1:25 for 16–18 h on a shaker set at 37 °C. Peptides were desalted using the Oasis µElution plate (Waters), speed vac-dried and reconstituted in 0.15% formic acid. For each sample, 200 ng of peptides were loaded onto Evotips (Evosep) following the manufacturer’s LC/MS–MS analysis protocol.

### Mass spectrometry data acquisition and protein identification

LC–MS/MS data for TCM and TAM samples was acquired respectively on the timsTOF HT mass spectrometer attached to a CaptiveSpray2 ion source (Bruker Daltonics) and timsTOF Pro2 mass spectrometer with a CaptiveSpray ion source (Bruker Daltonics). Both instruments were coupled to Evosep One (Evosep) LC equipped with a PepSep™ C18 column (8 cm long, 150 µm I.D., 1.5 µm particle size; Bruker Daltonics). Peptides were separated using the 60 samples per day (SPD) method, corresponding to a 21-min gradient at a constant flow rate of 1 µL/min, with the column maintained at 50 °C. MS spectra were recorded in positive ion dia-PASEF mode^63^ over a mass range of 100–1700 m/z with ion mobility (1/K0) ranging from 0.70 and 1.3 V-s/cm^2^, ramp time of 100 ms, and accumulation time 100 ms. Serial MS2 fragmentation was performed over 24 IM windows of 25 m/z isolation width with a cycle time of 1.38 s. The collision energy was dynamically ramped from 20 eV at ion mobility of 0.6 1/K0 to 52 eV at 1.6 1/K0. All data was acquired in two technical replicates.

DIA raw files were analysed using DIANN (v1.8.1), integrated into a cloud-based quantms pipeline^64^, against the UniProt *Homo sapiens* database (Swissprot, etc.). For the TCM samples, in addition to the Homo sapiens database, a common repository of FBS proteins^65^ was added as a contaminant to the search database. Protein identification was performed using default settings for fully tryptic peptides with up to two missed cleavages, a minimum of 6 and a max of 40 amino acids, and up to 3 modifications. Carbamidomethylation (C, + 57.0215 Da) was used as the fixed modification, while oxidation (M, + 15.999 Da) and protein N-terminal acetylation (+ 42.0106 Da) were set as variable modifications. Peptide and protein lists were filtered with a 1% False Discovery Rate (FDR), with MS1 and MS2 mass tolerances automatically determined by DIA-NN. The MaxLFQ algorithm^66^ embedded in DIA-NN was used for protein quantification, with retention time-based cross-run normalisation enabled. Biological annotation, subcellular localisation and functional enrichment for the differentially regulated proteins were evaluated using Uniprot, Gene Ontology and Ingenuity Pathway Analysis (IPA, Qiagen) databases.

### RNA sequencing and transcriptomic analysis

Macrophages were generated as outlined above and 1e6 cells were collected, centrifuged, supernatants removed and cell pellets were frozen on dry ice. Samples were stored at -80 until they were shipped to Azenta Genewiz for RNA extraction, library preparation and sequencing. FastQ files were then processed using the Illumina DRAGEN pipeline, software version 4.0 (07.021.645.4.0.3). Reads were mapped to the GRCh38 version of the human genome DRAGEN RNA-Seq spliced aligner (version of hash table dated 20,231,117, “sw_version”, ”01.003.044.4.0.3″, ”hash_table_version”: “8″) and quantified with enabled GC bias correction and automatic strand detection. Counts were then processed using the DESeq2 R package for differential expression and GSEA pathway enrichment analysis was performed using the HALLMARKs gene sets. Normalised log2-transformed CPMs were used for GSVA scoring of myeloid signatures. These scores were compared to the reference M-CSF treated samples using paired t-test based on donors, significance was defined by *p*-values < 0.05 after Benjamini-Hochberg (BH) multiple testing correction.

### Measurement of cellular energy metabolism

Seahorse metabolic flux analysis was used to determine the metabolic demands of cells. Glycolysis, OXPHOS and FAO were analysed using a Seahorse XF96 analyser (Agilent). Protocols were adapted from manufacturers recommendations. Seahorse cartridge plates were hydrated 24 h prior to use using Seahorse XF calibrant solution. Macrophages were harvested from culture plates and were seeded in seahorse assay plates at a density of 7.5e4 cells/well and left to adhere for 2 h at 37 °C.

Media was replaced with 180 ul/well Seahorse XF RPMI medium, supplemented appropriately for each assay (Table 1), and left at 37 °C for 30 min. Compounds were prepared in basal media at 10× concentration and sequentially injected according to the manufacturers user guide).

**Table 1:** Basal media supplements and concentrations of Seahorse injections.

Seahorse Kit (Agilent)	Basal media supplements	Injection A (20uL)	Injection B (22uL)	Injection C (25uL)	Injection D (27uL)
Mito stress test (103,015–100)	2 mM L-glumatine 1 mM Sodium Pyruvate	100 mM Glucose	15 μM Oligomycin	10 μM FCCP	5 μM Rotenone/Antimycin A
Palmitate-BSA FAO (103,693–100)	2 mM XF Glucose 0.5 mM L-Carnitine	40 μM Etomoxir	15 μM Oligomycin	10 μM FCCP	5 μM Rotenone/Antimycin A

Glycolysis (mpH/min) was calculated by subtracting the last rate measurement before Glucose injection (timepoint 4) from the maximum rate before Oligomycin injection (timepoint 8). Maximal respiratory capacity (MRC) pmol/min was calculated by subtracting the OCR values after adding rotenone/antimycin A from values after adding FCCP. Data were analysed in WAVE and plotted using GraphPad Prism.

### Cytokine analysis

Supernatants from the T-cell suppression assay were assessed for IFNγ release (R&D #DY285B) per the manufacturer’s protocol, with supernatants diluted 1 in 4. The plates were read using the Envision with a Europium readout.

### Statistics

Statistical analysis was carried out with the GraphPad Prism software; error bars show ± SEM. Statistical differences among groups were determined using student’s t-test, one-way ANOVA, or two-way ANOVA analysis. Statistical significance was determined as follows: *p < 0.05, **p < 0.001, ***p < 0.0001, and ****p < 0.00001. For whole-cell proteomics and secretomic analysis, normalised protein intensities from DIA-NN were averaged from two technical replicates. Welch’s t-test, with a permutation-based FDR, was performed on log2-transformed average LFQ intensities using the Perseus software platform^67^. Protein ratios showing a 0.5-fold change with a *p* value of ≤ 0.05 were considered differentially expressed proteins. For functional analysis and biological enrichment of significant differentially regulated proteins, Gene Ontology (GO) terms were referenced using QuickGO (https://www.ebi.ac.uk/QuickGO) and gene lists for specific GO terms were downloaded from Uniprot (https://www.uniprot.org/). Ingenuity Pathway Analysis (IPA, Qiagen) was used for pathway enrichment analysis.

## Supplementary Information

Supplementary Information.

## Data Availability

The proteomics dataset generated and/or analysed during the current study is available in the MassIVE repository, under the accession number PXD063897. RNA seq data has been deposited in the NCBI Gene Expression Omnibus (GEO) database and are publicly available as of the date of publication with the accession number GSE297659.
