# Supplementary material for: Characterisation of human in vitro tumour-associated macrophage models to define translational relevance
Source: Sci Rep. 2025 Nov 28;15:45648. doi: 10.1038/s41598-025-30224-w (PMC12753636; doi:10.1038/s41598-025-30224-w)
Supplement: Supplementary file 1 — Supplementary Information. [file 41598_2025_30224_MOESM1_ESM.pdf]

Supp Table 1: Details of the immortalised cell lines used for TCM generation

Cell Line Name	Parent Species	Containment for Growth:	Source	Source Part Number	Cell Line Type	Source Tissue	Parent Disease Origin Tissue	Parent Disease	Gender	Ethnicity Type:	Cell Type:	Growth Type:
MDA-MB-231	Human (Homo sapiens)	Level 1	ATCC	HTB-26	Mammalian Cell Line	Pleural fluid/effusion	Breast/Mammary Gland	Adenocarcinoma (not specified)	Female	Caucasian (White)	Epithelial	Adherent
PANC-1	Human (Homo sapiens)	Level 1	ATCC	CRL-1469	Mammalian Cell Line	Pancreas	Pancreas	Carcinoma (not specified)	Male	Caucasian (White)	Epithelial	Adherent
SK-OV-3	Human (Homo sapiens)	Level 1	ATCC	HTB-77	Mammalian Cell Line	Ovary/Ovarian	Abdomen/Ascites	Adenocarcinoma (not specified)	Female	Caucasian (White)	Epithelial	Adherent
786-O	Human (Homo sapiens)	Level 1	ATCC	CRL-1932	Mammalian Cell Line	Kidney	Kidney	Renal Cell Cancer (RCC)	Male	Caucasian (White)	Epithelial	Adherent
Hep 3B2	Human (Homo sapiens)	Level 2 (Cells Contains Hepatitis B)	ATCC	HB-8064	Mammalian Cell Line	Liver	Liver	Hepatocellular Cancer (HCC)	Male	Black	Epithelial	Adherent
DU 145	Human (Homo sapiens)	Level 1	DSMZ	ACC-298	Mammalian Cell Line	Prostate	Prostate	Prostate Cancer	Male	Caucasian (White)	Epithelial	Adherent
JIMT-1	Human (Homo sapiens)	Level 1	DSMZ	ACC-589	Mammalian Cell Line	Pleural fluid/effusion	Breast/Mammary Gland	Ductal Carcinoma InSitu (DCIS) (Breast Cancer)	Female	Caucasian (White)	Epithelial	Adherent

Supplemental Table 2: Details of the primary ascites fluid donors

Ascites Donor Number	Patient ID	Primary Diagnosis	Draw Date	Patient Sex	Patient Race	Patient Age At Collection	Patient Height (cm)	Patient Weight (kg)	Patient Tobacco History	Patient Alcohol History
Ascites Donor 1	130898221	Ovarian Cancer, Serous Carcinoma	3/13/2023	Female	White	63	165	64.86	Never Used	No Use
Ascites Donor 2	130873774	Ovarian Cancer, Serous Carcinoma	02/02/2023	Female	White	65	156.01	65.32	Current Cigarette	No Use
Ascites Donor 3	130873770	Ovarian Cancer, Papillary Serous Carcinoma	1/26/2023	Female	White	72	180.01	80.29	Never Used	No Use
Ascites Donor 4	200037966	Ovarian Cancer, Serous Carcinoma	1/13/2023	Female	White	63	180.34	115.21	Never Used	No Use
Ascites Donor 5	130845443	Ovarian Cancer, Serous Carcinoma	1/19/2023	Female	White	63	168	66.68	Never Used	No Use
Ascites Donor 6	130845439	Ovarian Cancer, Serous Carcinoma	3/17/2023	Female	White	72	158.24	55.34	Never Used	No Use
Ascites Donor 7 (1)	130873768	Ovarian Cancer, Papillary Serous Carcinoma	02/08/2023	Female	White	67	159.99	82.55	Never Used	No Use
Ascites Donor 8 (2)	130882907	Ovarian Cancer, Serous Carcinoma	1/18/2023	Female	White	73	170	59.87	Never Used	No Use
Ascites Donor 9 (3)	130860122	Ovarian Cancer, Serous Carcinoma	03/10/2023	Female	White	75	160.02	70.31	Never Used	No Use
Ascites Donor 10 (4)	130890102	Ovarian Cancer, Papillary Serous Carcinoma	02/10/2023	Female	White	75	154.99	66.68	Never Used	No Use

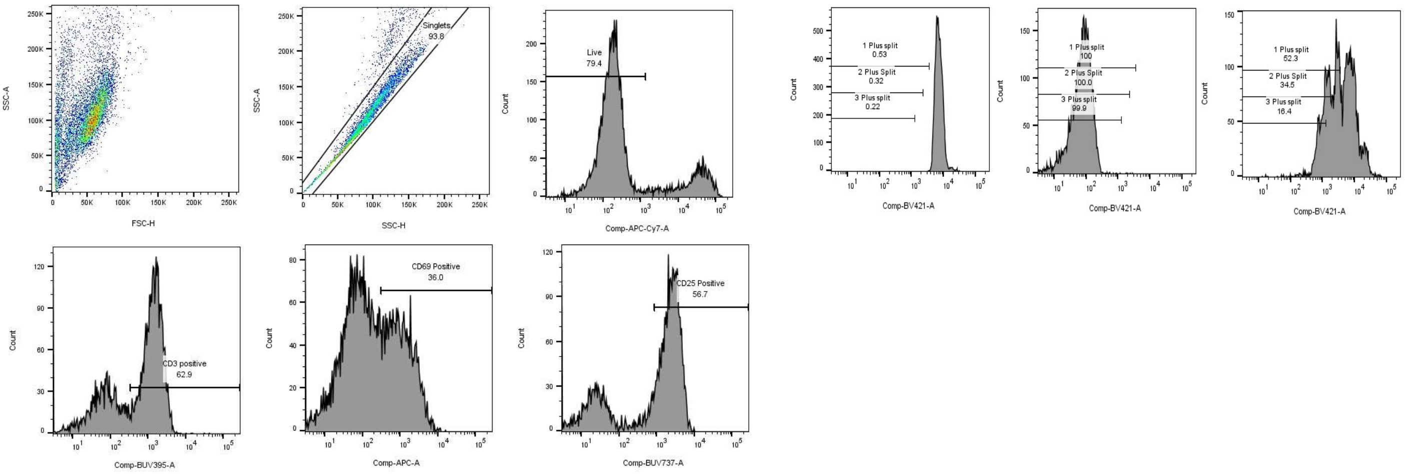

Supp Fig. 1. Gating strategy for flow cytometry analysis.

Cells were harvested using a non-enzymatic cell dissociation solution, stained using a NiR live/dead dye and resuspended in flow buffer (PBS, 2% BSA, 0.1% Sodium Azide, 2mM EDTA) supplemented with Fc block (InnovexBio). Cells were stained with anti-CD4 (PE), anti-CD3 (BUV395), anti-CD8 (BV650), anti-cD25 (BUV737), and anti-CD69 (BV421). Cells were then fixed and analysed by flow cytometry. Cellular debris was excluded through gating on the bulk population using FSC-A/SSC-A, single cells were identified using FSC-A/FSC-H gating, and live cells were identified by gating on the live/dead- population (APC-Cy7 channel). Cells were identified based on cell surface markers with negative controls as reference. T cell proliferation was determined through analysing peak shifts in the BV421 channel. Monocytes from healthy donor PBMCs were differentiated over 7 days in media + 100nG/mL M-CSF with a replenishment of culture media + 100nG/mL M-CSF on day 3. Donor matched CD3+ T-cells were co-cultured for 4 days with differentiated macrophages.

Supp Fig. 2. Phenotypic and functional characterisation of hMDMs exposed to MDA-MB-231 TCM compared with IL-4/IL-13 cytokines and additional analysis of T cells co-cultured with TCM-polarised hMDMs.

Monocytes from healthy donor PBMCs were differentiated over 7 days in media + 50nG/mL M-CSF with a replenishment of culture media + 50nG/mL M-CSF on day 3. M0 macrophages were cultured in 50nG/mL M-CSF. MDA-MB-231 TCM macrophages were cultured in 1:1 MDA-MB-231 TCM:complete media + 50nG/mL M-CSF. IL-4/IL-13 macrophages were differentiated as M0 macrophages with 20nG/mL IL-4 and IL-13 for the final 24 hours. Donor matched CD3+ T-cells were co-cultured for 4 days with differentiated macrophages.

A) CD86, CD163 and CD206 expression normalised to 50nG/mL M-CSF differentiated macrophages assessed by flow cytometry. B) T-cell proliferation and matched IFNy release from a T-cell and macrophage co-culture using CD3/CD28 stimulation. C) Effect of TCM-polarised hMDMs on early T cell activation marker CD69 in CD3+ T-cells 4 days post-CD3/CD28 stimulation and co-culture with TCM-hMDMs (macrophage:T cell = 1:2).

Each point represents one donor, values shown as Mean +/- SEM. Statistical significance calculated by one-way ANOVA with Dunnet’s multiple comparisons test and shown where * p-value < 0.05, ** p-value < 0.005 and *** p-value < 0.0005 and **** p-value < 0.0001.

A)

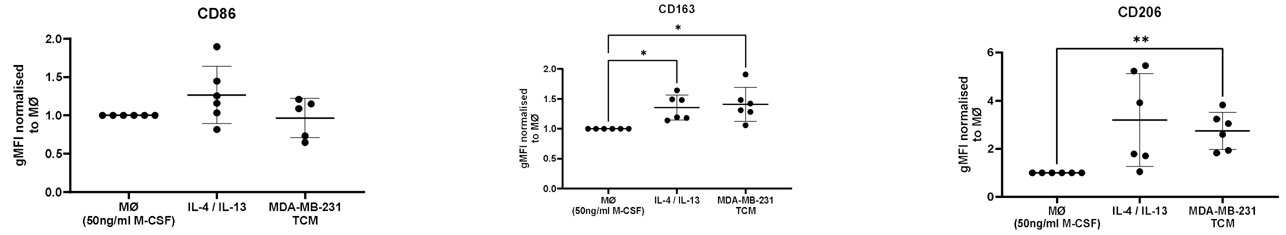

B)

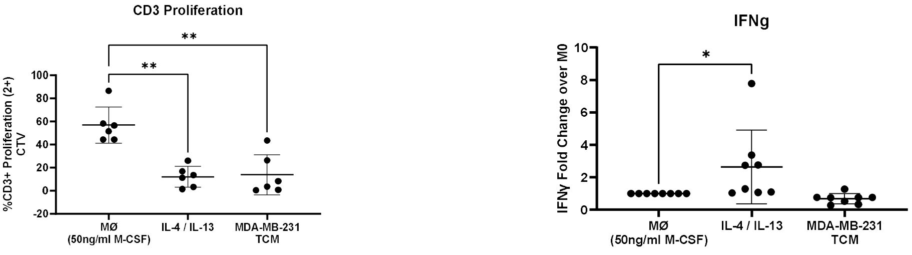

C)

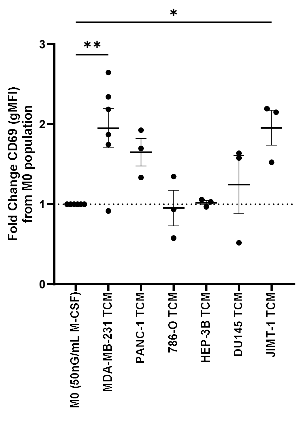

Supp Fig. 3.

Full ECAR and OCR values of following the Seahorse flux analysis with Mito Stress Test and injection of glucose. Graphs show all conditions for individual donors, including time points and injections. Monocytes from healthy donor PBMCs were differentiated over 7 days in complete media + 50nG/mL M-CSF with a replenishment of culture media + 50nG/mL M-CSF on day 3 for M0 macrophages. All TCM polarised macrophages from healthy donor PBMCs were cultured in TCM:RPMI 1:1 containing 50nG/mL M-CSF with replenishment of media at day 3.

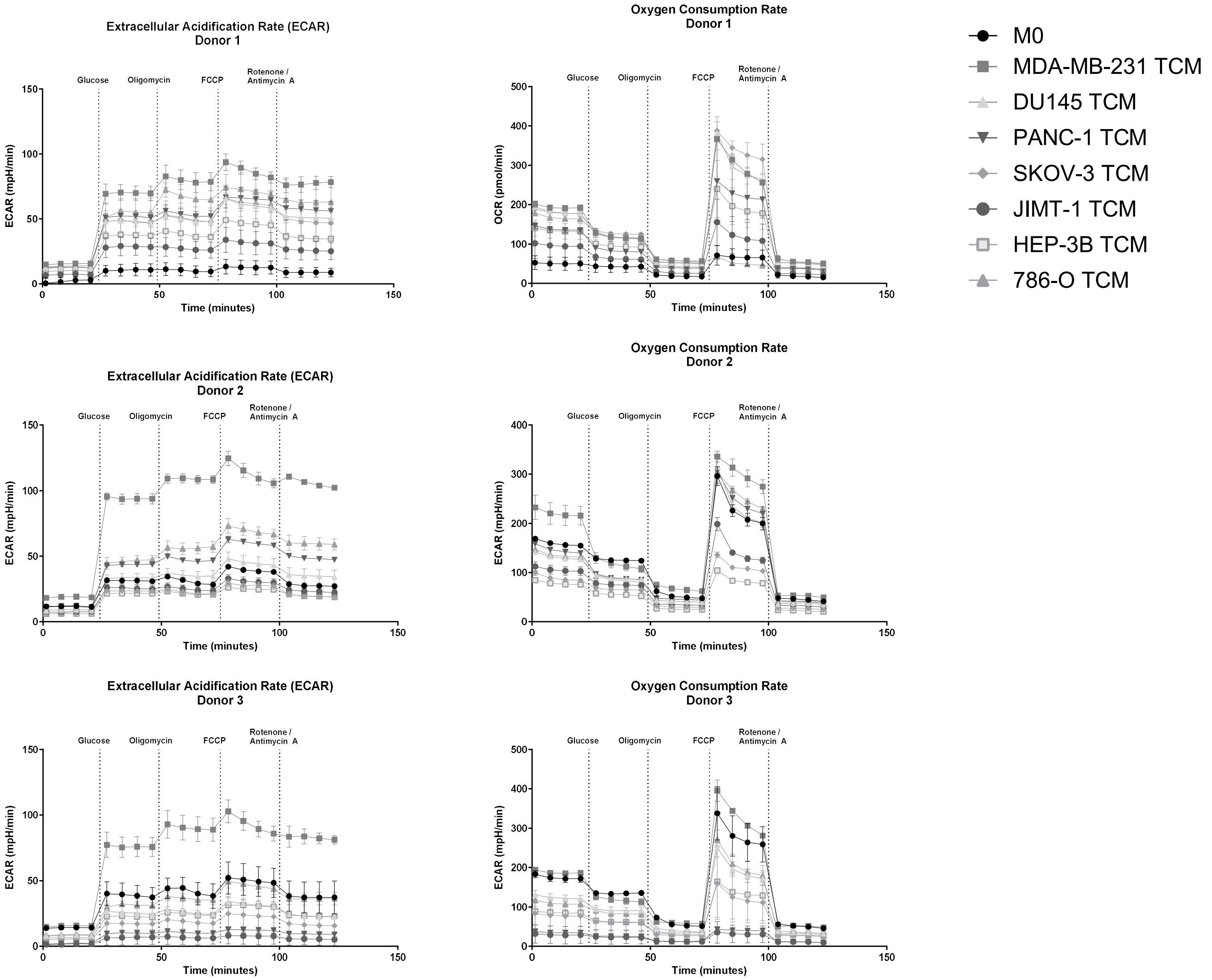

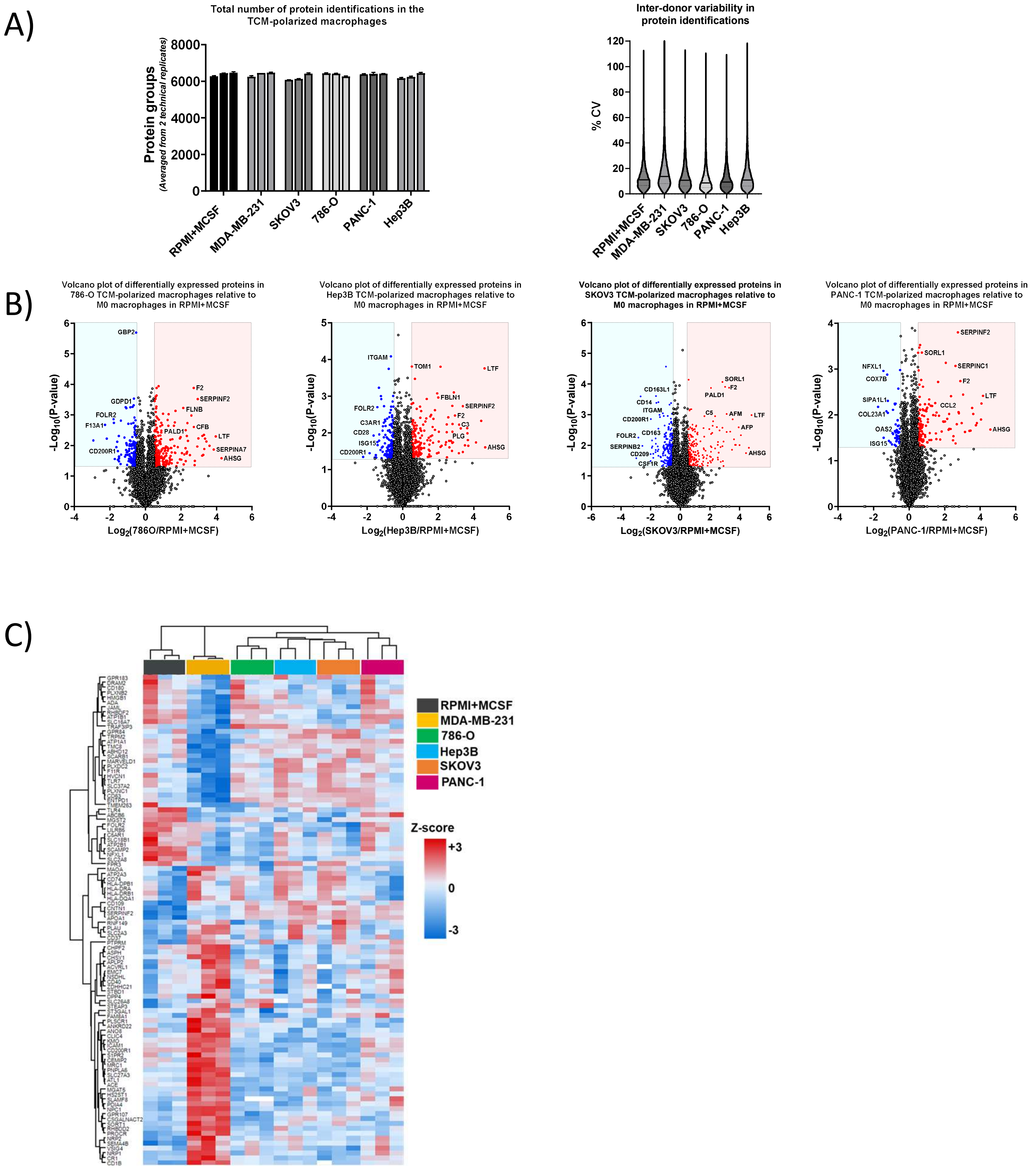

Supp Fig. 4. Summary of identification of proteins by proteomic analysis.
A) Number of proteins identified for each cell sample and inter-donor variability. B) Volcano plot displaying all differentially expressed proteins. Proteins highlighted in red were significantly increased and those in blue were significantly decreased. C) Heatmap of differentially expressed cell surface proteins induced by polarisation conditions analysed with respect to top differentially expressed proteins induced by MDA-MB-231 TCM. Monocytes from healthy donor PBMCs were differentiated over 7 days in complete media + 50nG/mL M-CSF with a replenishment of culture media + 50nG/mL M-CSF on day 3 for “RPMI+MCSF” macrophages. All TCM polarised macrophages from healthy donor PBMCs were cultured in TCM:RPMI 1:1 containing 50nG/mL M-CSF with replenishment of media at day 3.

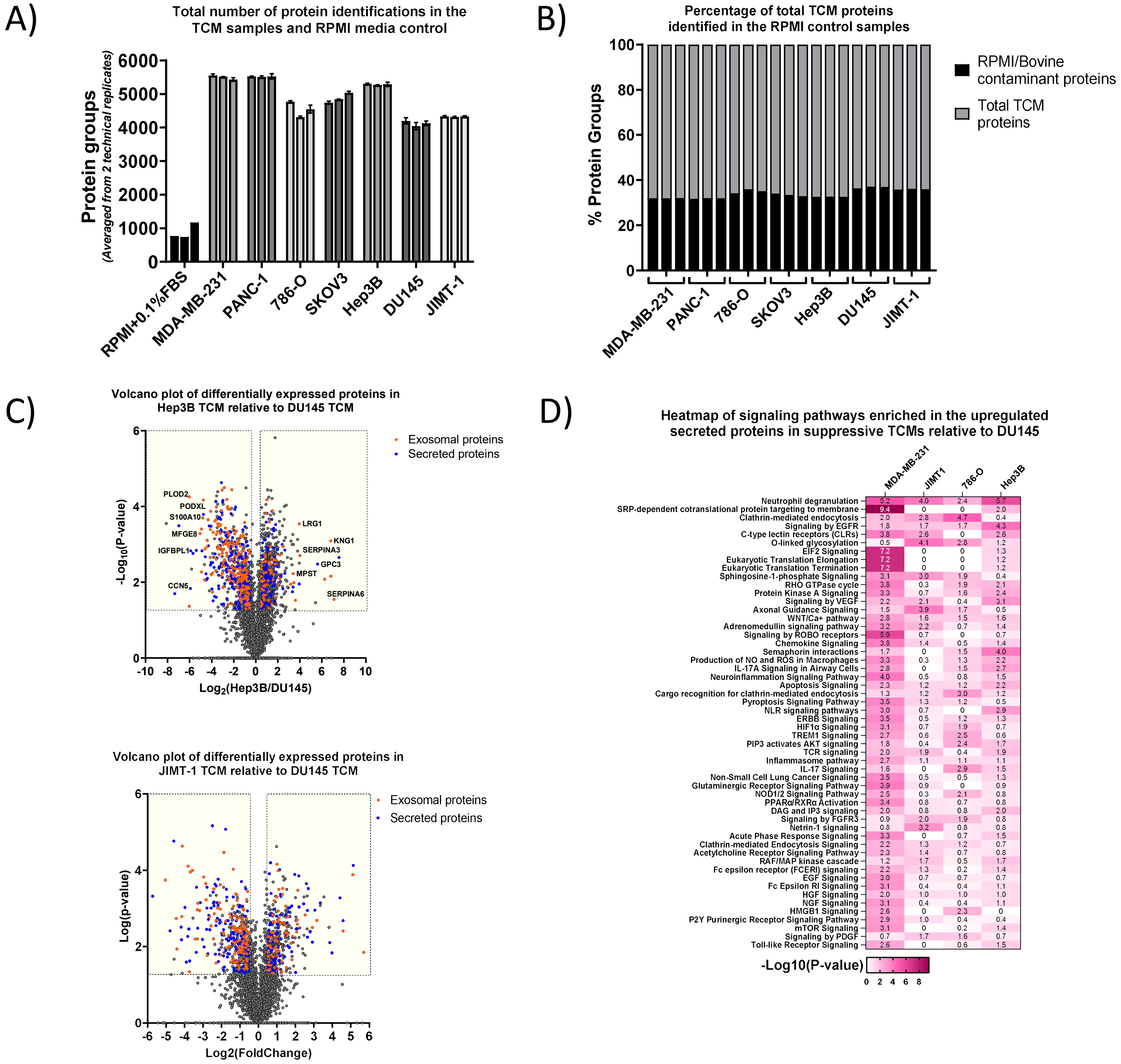

Supp Fig. 5. Summary of secretomics proteins identified.

A) Number of proteins identified in RPMI and the tumour-conditioned media sample analysed with LC-MS/MS. B) Number of bovine and RPMI contaminant proteins identified in each sample. C) Volcano plot representing differentially expressed proteins in Hep3B and JIMT-1 relative to DU145 TCM. Proteins highlighted in blue are exosomal proteins and proteins highlighted in orange are secreted proteins. D) Heatmap showing significantly enriched pathways specifically for proteins annotated as "secreted" and with increased expression in suppressive TCMs compared to non-suppressive DU145 (p-value cutoff ≤0.05).

Supp Fig. 6.

A) Full ECAR and OCR values following the Seahorse flux analysis Fatty Acid Oxidation test and palmitate substrate. Graphs show all conditions for individual donors, including time points and injections. B) Metabolic profile of lipid-depleted cells pre- and post- etomoxir addition. Monocytes from healthy donor PBMCs were differentiated over 7 days in complete media + 50nG/mL M-CSF with a replenishment of culture media + 50nG/mL M-CSF on day 3 for M0 macrophages. MDA-MB-231 TCM polarised macrophages from healthy donor PBMCs were cultured in TCM:RPMI 1:1 containing 50nG/mL M-CSF with replenishment of media at day 3.

A)

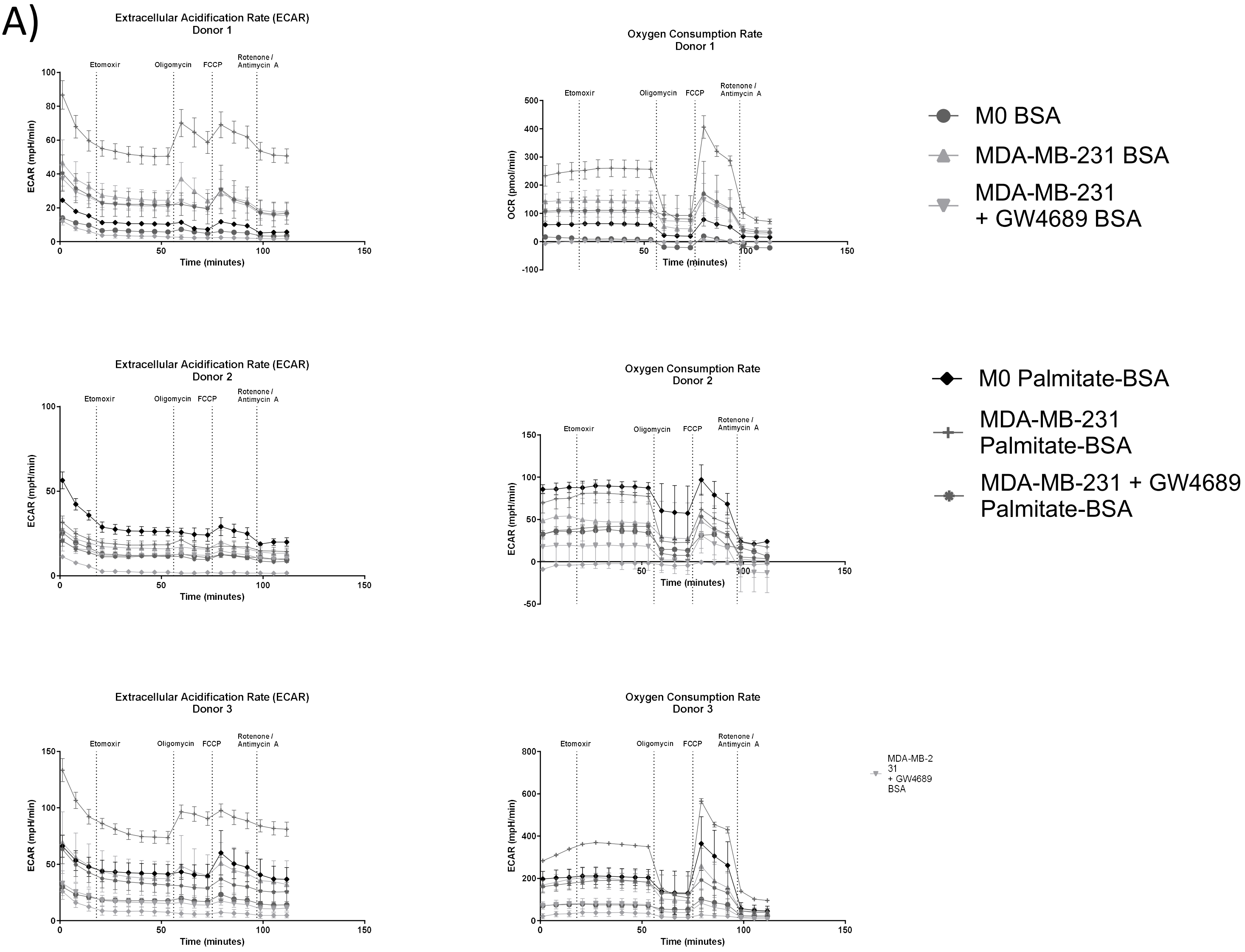

B)

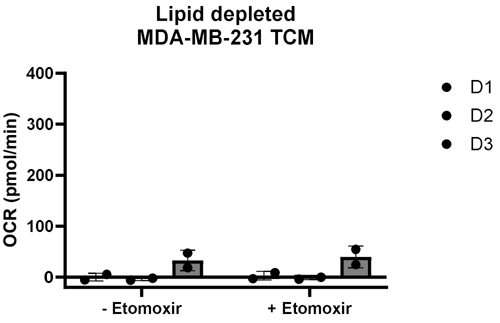

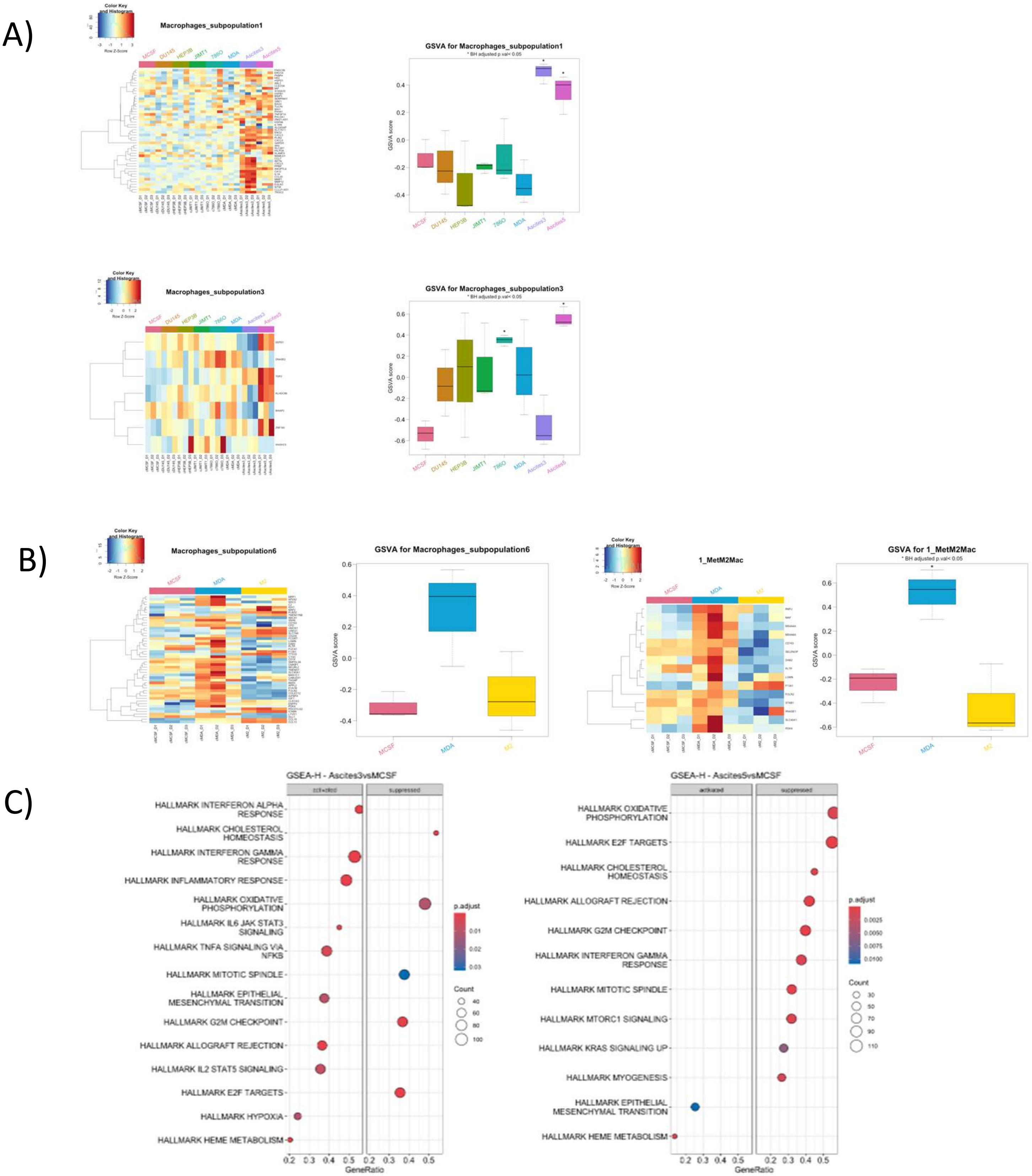

Supplementary Fig. 7.

Monocytes from healthy donor PBMCs were differentiated over 7 days in media + 50nG/mL M-CSF with a replenishment of culture media + 50nG/mL M-CSF on day 3. “MCSF macrophages” were cultured in 50nG/mL M-CSF. MDA-MB-231 TCM macrophages were cultured in 1:1 MDA-MB-231 TCM:complete media + 50nG/mL M-CSF. “M2” macrophages were differentiated as M0 macrophages with 20nG/mL IL-4 and IL-13 for the final 24 hours.

A) Heatmaps of scaled log2-transformed gene expression data across conditions from published TAM signatures. Boxplots of GSVA scores per sample of published TAM signatures. * Benjamini-Hochberg (BH) corrected p-values < 0.05 compared to MCSF. B) Heatmaps and boxplots showing GSVA scores for M-CSF, IL-4/IL-13 or MDA-MB-231 TCM polarisation conditions compared to published TAM signatures. C) GSEA dotplots of top enriched and suppressed pathways for HALLMARK gene sets in differentially exprressed genes in each ascites fluid samples vs MCSF controls.
